# Dry side of the core: a meta-analysis addressing the original nature of the ABA signalosome at the onset of seed imbibition

**DOI:** 10.3389/fpls.2023.1192652

**Published:** 2023-07-05

**Authors:** Guillaume Née, Thorben Krüger

**Affiliations:** Institute for Plant Biology and Biotechnology, University of Münster, Münster, Germany

**Keywords:** abscisic acid (ABA), seed germination, protein post-translational modifications (PTMs), molecular signalling, proteomics

## Abstract

The timing of seedling emergence is a major agricultural and ecological fitness trait, and seed germination is controlled by a complex molecular network including phytohormone signalling. One such phytohormone, abscisic acid (ABA), controls a large array of stress and developmental processes, and researchers have long known it plays a crucial role in repressing germination. Although the main molecular components of the ABA signalling pathway have now been identified, the molecular mechanisms through which ABA elicits specific responses in distinct organs is still enigmatic. To address the fundamental characteristics of ABA signalling during germination, we performed a meta-analysis focusing on the Arabidopsis dry seed proteome as a reflexion basis. We combined cutting-edge proteome studies, comparative functional analyses, and protein interaction information with genetic and physiological data to redefine the singular composition and operation of the ABA core signalosome from the onset of seed imbibition. In addition, we performed a literature survey to integrate peripheral regulators present in seeds that directly regulate core component function. Although this may only be the tip of the iceberg, this extended model of ABA signalling in seeds already depicts a highly flexible system able to integrate a multitude of information to fine-tune the progression of germination.

## Introduction

During evolution, the emergence of desiccation-tolerant seeds (orthodox seeds) within the green *taxa* appears to be an outstandingly efficient propagative strategy, as most of the higher plants reproduce trough the production of seeds (Baroux and Grossniklaus, 2019). Dry seeds are quiescent and some of the most resilient known biological structures, serving as vehicle and vault for plant genetic material. Because germination, which is marked by the emergence of the radicle over surrounding structures, is associated with the loss of stress tolerance and mobility traits, the presence of environmental stressors (i.e. temperature, water, or oxygen limitations) prevents sprouting; seeds only complete their germination if surrounding conditions are favourable for further vegetative growth.

Nevertheless, water uptake in stress-free environments does not systematically lead to germination completion. In fact, the absence of germination upon imbibition of viable seeds under optimal conditions is referred to as “seed dormancy*”* (Finch-Savage and Leubner-Metzger, 2006; Soppe and Bentsink, 2020). This specific trait prevents germination of freshly settled seeds during ephemerally favourable conditions to ensure that seedlings are established within the optimal growth season. Seed dormancy provides time for seed dispersion after being shed from the mother plant and contributes to soil seed bank maintenance. Dormancy is established during seed maturation, and its alleviation occurs gradually with time or after specific environmental *stimuli* that inform seeds about drastic changes in the environment. For example, stratification (i.e. sustained seed imbibition at low temperature) signals the experience of a cold season and leads to a reduction of dormancy levels in many spring-sprouting species (Allen and Meyer, 1998; Forbis, 2010; Baskin and Baskin, 2014). Similarly, exposure to smoke-derived compounds (i.e. karrikins) breaks dormancy, as it signals there will be less competition for resources after a land fire (Pausas and Lamont, 2022).

The developmental transition from seed to sprout is characterized by sequential check points and molecular switches (Fait et al., 2006; Galland et al., 2014; Basbouss-Serhal et al., 2015; Bai et al., 2017; Chahtane et al., 2017; Bai et al., 2018). According to internal signals (i.e. dormancy) or external signals (i.e. occurrence of unfavourable conditions for growth), seeds pause germination progression to retain their protective features. This “stop or go” mechanism determines the time and location of seedling emergence and, hence, the conditions within which a plant will establish itself and its reproductive success.

Many stress and developmental responses are controlled by the phytohormone abscisic acid (ABA). The physiological concentration of ABA is tightly regulated by the balance between its synthesis and degradation (Nambara and Marion-Poll, 2005) or the conjugation and release from its storage form, ABA glucose ester (Lee et al., 2006; Xu et al., 2012; Liu et al., 2015). The essential role of ABA in germination repression was discovered over 50 years ago (Sondheimer et al., 1968), and germination readouts have helped identify genes involved in ABA metabolism and signalling (Koornneef et al., 1982; Karssen et al., 1983; Koornneef et al., 1984; Finkelstein, 1994). However, its mainstream signalling pathway was only fully elucidated in 2009 with the discovery of ABA receptors.

Changes in ABA homeostasis are perceived in the cell by a group of soluble proteins from the StART (Steroidogenic Acute Regulatory—StAR—Related lipid Transfer) family named pyrabactin resistance (PYR), PYR-like (PYL) or Regulatory Component of ABA Receptors (RCARs), according to the screens from which they were identified (Ma et al., 2009; Park et al., 2009). When ABA binds to RCAR, this leads the receptor to form a stable tertiary complex with type 2C protein phosphatases from clade A (PP2CAs). As the RCAR-PP2CA interaction hinders the PP2CA catalytic site, this consequently prevents dephosphorylation of downstream targets such as SNF1-related protein kinases of group 2 (SnRK2s). SnRK2 kinases are central components of plants’ stress signal transduction (Kulik et al., 2011), and their full activation requires phosphorylation on specific residues located in the “activation loop” (Ng et al., 2014). Thus, when RCAR complexes with PP2CAs and prevents them from dephosphorylating the kinase activation loop, SnRK2s activate ABA responses by trans-phosphorylating a large array of targets *in vivo* (Shin et al., 2007; Wang et al., 2013). Downstream transcriptional ABA responses involve several sequence-specific DNA binding proteins such as group A basic zipper transcription factors (bZIPs), like ABA-insensitive 5 (ABI5), for which phosphorylation by SnRK2s kinases on precise residues enhances transcriptional activity (Yu et al., 2015). In addition to SnRK2 inhibition, an efficient shut-off of ABA responses can be achieved by PP2CAs, which can directly dephosphorylate bZIPs (Antoni et al., 2012; Lynch et al., 2012). Alongside the mainstream ABA signalling pathway exist a variety of molecular systems involved in quantitative and qualitative tuning of ABA responses by targeting core components that have been described within the last 10 years (Ali et al., 2020; Lim et al., 2022). Still, how ABA elicits specific responses according to distinct *stimuli*, developmental and cellular contexts is not completely understood.

The scope of this manuscript is to offer new perspectives about the flexibility of ABA responses from a singular point of view: the dry seed. Using available scientific resources from Arabidopsis (*Arabidopsis thaliana)* research, we performed a meta-analysis to redefine the nature and the functioning of the ABA core signalosome in seeds. To map the initial wiring of the core ABA signalosome in seeds, we integrated state-of-the-art proteome information with interactome data and results from functional studies combining core components in reconstituted systems *in vivo*. Further, we incorporated proteins already present in dry seeds that directly modulate core component functions over these central molecular chassis. Finally, we propose an extended model of ABA signalling during early seed imbibition depicting the routes and the management of information fluxes within the system to illustrated how ABA responses can be tuned in a quantitative and qualitative manner to adjust the progression of germination.

## Methods

### Data collection and analysis

Intensity-based absolute quantification (iBAQ) values were used to define the relative abundances in *Arabidopsis thaliana* (Col-0) dry seeds and were obtained from the study by Mergner et al., 2020. Heat maps were built using heat mapper (http://www.heatmapper.ca/) (Babicki et al., 2016). Quantitative data for the inhibition of PP2CAs by RCARs and the inhibition of SnRK2s by PP2CAs were obtained from the study by Tischer et al., 2017 and Ruschhaupt et al., 2019, respectively. The ABA concentrations required to inhibit germination of 50% of a seed batch ([ABA] IC_50_) and the relative transgene expression levels in lines overexpressing *RCARs* were taken from the study by Yang et al., 2016. Protein-protein interactions for the 7 seed PP2CAs were retrieved from Biogrid (https://thebiogrid.org/) and IntAct (https://www.ebi.ac.uk/intact/home) repositories on 20 March 2023. Interaction partner overlaps were analysed using FLAME (https://bib.fleming.gr:8084/app/flame) (Thanati et al., 2021).

### Literature search and selection

To collect information about regulation of the ABA core signalosome at the protein level, we searched PubMed (https://pubmed.ncbi.nlm.nih.gov/) for relevant literature using combinations of key words for each core signalling component AND *Arabidopsis* AND *regulation* OR *inhibition* OR *activation* OR *degradation* OR *phosphorylation* OR *ubiquitinoylation* OR *post translational modification*. Among hundreds of responses, we further manually filtered relevant research articles using the following criteria: (i) Experiments demonstrating a direct interaction were presented and properly controlled, (ii) the functional consequences of the interaction were investigated, and (iii) the effects of genetic null mutation and/or overexpression were investigated in response to an ABA-related trait (i.e. germination or stress tolerance).

### Analyses of protein sequence alignments, phylogenetics, and protein structures

Protein amino acid sequence alignments of PP2CAs were made using the ClustalOmega algorithm of MegaAlign. Phylogenetic trees for the different ABA core signalosome components were constructed with MegaAlign using the neighbour joining algorithm based on ClustalOmega alignments of full protein sequences obtained from UniprotKB. Protein accession numbers are listed in **Supplementary Table S1**. Structures of Arabidopsis ABA-bound RCAR14 and ABA-bound RCAR1 in complex with HAB1 were obtained from the PDB repository (https://www.rcsb.org/) with the accession number 3KB3 and 3OQU, respectively. Structural modelling of AHG1 was obtained from the Alphafold repository (https://alphafold.ebi.ac.uk/). Superimposition of protein structures was performed using PDBeFold (https://www.ebi.ac.uk/msd-srv/ssm/). Visualization and analyses were performed using PyMOL software (DeLano Scientific LLC).

## Results

### Starting deal: composition of ABA mainstream signalling cascade in dry seeds

**Figure 1:**
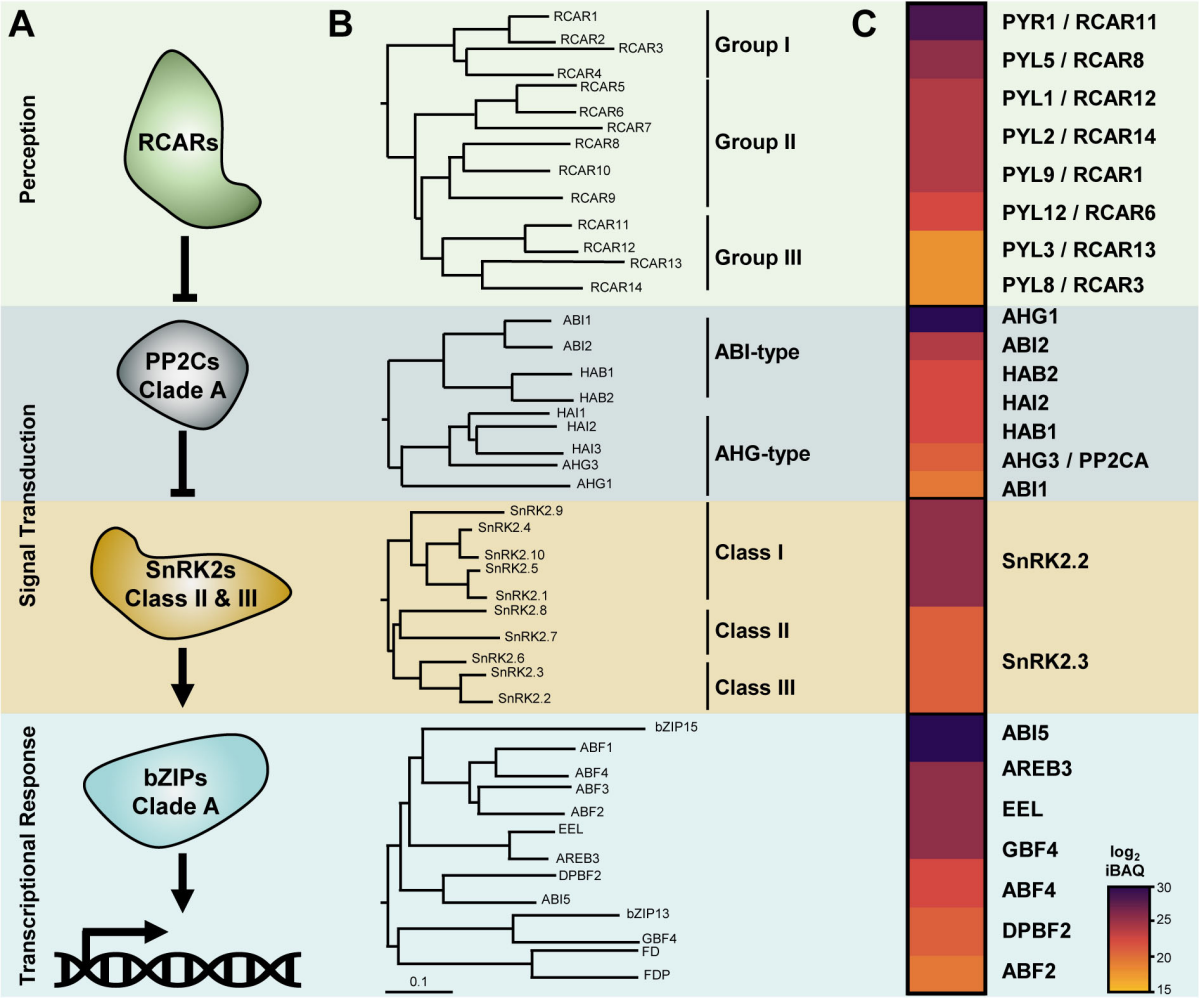
Composition of ABA core signalling pathway in Arabidopsis dry seeds. **(A)** ABA receptors (RCAR) repress clade A phosphatase 2C (PP2CAs) to relieve SNF1-related protein kinases 2 (SnRK2s) from their inhibition. Active SnRK2s phosphorylate bZIP transcription factors from group A to induce ABA transcriptional responses. **(B)** Phylogenetic trees of RCARs, PP2CAs, SnRK2s, and group A bZIP proteins from *Arabidopsis thaliana*, TAIR10 (Col-0). Denominations for closely related isoforms are marked on the right. **(C)** Composition of the ABA signalosome equipment in dry seed of *Arabidopsis thaliana* (Col-0). Log_2_ transformed intensity-based absolute quantification (iBAQ) values corresponding to the relative protein abundances of each component were obtained from the study by Mergner et al., 2020, and presented as a heat map where dark tones indicates higher accumulation.

The core ABA signalling pathway involves the relay between the ABA receptors (RCARs), the clade A PP2C, and the class II/III SnRK2 kinases (**Figure 1A**). In seed plants, each level of the ABA core signalosome is encoded by a multigenic family. The Arabidopsis genome encodes 14 ABA receptors, 9 clade A PP2Cs, 10 SnRK2 kinases, and 13 group A bZIPs. Phylogenetic analyses of the amino acid sequences allow for further classification of isoforms in distinct subgroups within the families (**Figure 1B**). RCARs cluster in three groups: RCAR1-4 (group I), RCAR5-10 (group II), and RCAR11-14 (group III). PP2CAs divide in two subgroups, the ABA-Insensitive (ABI) type with 4 members, namely ABI1 and ABI2 and their respective homologues HAB1 and HAB2, and the ABA-Hypersensitive Germination (AHG) type, which includes AHG1, AHG3, Highly ABA Induced 1 (HAI1), HAI2, and HAI3 (Fuchs et al., 2013). Although AHG1 is related to the other clade A PP2Cs (from herein called canonical PP2CAs), it is the most divergent isoform of the AHG-type, and it is often considered a distinct evolutionary branch (Cutler et al., 2010; Tischer et al., 2017). Arabidopsis SnRK2 kinases comprise three distinct classes: class I, which includes SnRK2.1, SnRK2.4, SnRK2.5, SnRK2.9, and SnRK2.10; class II, which includes SnRK2.7 and SnRK2.8; and class III, which includes SnRK2.2, SnRK2.3, and SnRK2.6. Except for SnRK2.9, all SnRK2s are activated by osmotic stress, but only class III and to a lesser extent class II are activated by ABA (Maszkowska et al., 2021). Within group A bZIPs, the closest homologue of ABI5 is DC3 Promoter-Binding Factor 2 (DBPF2/bZIP67). These two bZIPs are related to a distinct branch that includes the ABA Binding Factors (ABF) 1 to 4, Enhanced Em Level (EEL) and ABA Responsive Element Binding 3 (AREB3). The bZIP group A also includes G-Box Binding Factor 4 (GBF4) and bZIP15, whose roles in ABA signalling are unclear, and FD and FD-Paralogue (FDP), which possibly arose from a function in ABA signalling during germination but have evolved towards the control of other processes (Romera-Branchat et al., 2020). The existence of multiple members at each step of the signalling relay raises the fundamental question of functional redundancy or specialization between the different isoforms. Addressing the relative contribution of each component within the ABA pathway is essential to understand how seeds integrate multiparameter signals to control the progression of germination. To this aim, it is necessary to qualitatively and quantitatively characterize the subset of ABA signalling components that are present in seeds and to address their network connectivity.

Nowadays, the availability of Arabidopsis tissue-specific proteomic data sets that include seed samples allows one to search for the presence and relative abundance of proteins involved in ABA signalling in a particular physiological context (Xiang et al., 2016; Zhang et al., 2019; Bassal et al., 2020; Mergner et al., 2020; Lynch et al., 2022). With 12,392 quantified proteins in the dry seed, data from the Arabidopsis Proteome Atlas currently provide the deepest view on the relative protein abundances in this organ (Mergner et al., 2020; Tena, 2020). Based on these data, we reconstituted the actual composition of the ABA signalosome in dry seed (**Figure 1C**). Eight out of the 14 receptors have been detected in dry seeds, including the four members of group III and two isoforms of group I and II. RCAR11 and RCAR8 appear to be the most abundant, and RCAR13 and RCAR3 the least abundant. At the exception of HAI1 and HAI3, all PP2CAs have been experimentally detected in dry seeds, and AHG1 is the most abundant. Class II SnRK2s were not detected in seeds; thus, only two ABA-responsive kinases, namely SnRK2.2 and SnRK2.3, make up the seed core ABA signalling machinery. In addition, seven group A bZIPs are present in dry seeds, of which ABI5 is the most abundant.

The focus brought by the Arabidopsis Proteome Atlas, regarding the composition at the protein level of the ABA core signalosome in dry seeds clearly reflects what has been found at the level of genetic data. For example, the quintuple *rcar13 2 1 5 6* mutant, in which most of the seed-abundant RCARs (i.e. RCAR11 and 8) are functional, shows a wild type (WT)-like ABA sensitivity during germination (full inhibition of germination at sub-µM ABA levels). In contrast, the quintuple *rcar3 8 10 11 14* mutant, retaining only three functional receptors of low abundance in the native context (RCAR1, 12, and 13), displays an ABA-blind phenotype during seed imbibition (germination rate >50% at concentrations on the scale of 100 µM) (Gonzalez-Guzman et al., 2012). Seeds of the single *ahg1* mutant possess elevated dormancy and the highest ABA sensitivity in comparison to other single *pp2ca* mutants, highlighting that the most abundant seed PP2CA, namely AHG1, is critical for promoting germination (Nishimura et al., 2007; Bhaskara et al., 2012; Née et al., 2017a). Similarly, seeds of single *ahg3* mutants also display an increased dormancy and ABA hypersensitivity during germination, suggesting that although AHG3 is one of the least abundant PP2CAs in seeds, it holds specific and critical functions that do not fully overlap with other members (Yoshida et al., 2006). Single mutations of the most abundant ABI-type PP2CA, namely ABI2, do not affect germination traits, as the *abi2* loss-of-function mutant shows a WT-like ABA sensitivity during germination. Nevertheless, the double *abi1 2* mutant or triple *abi1 2 hab1* mutant, such that only the activity of HAB2 is retained, display an increased ABA sensitivity in seed germination assays (Rubio et al., 2009). The triple *hai1 2 3* mutant shows a reduced and not an increased ABA sensitivity during germination, suggesting that these PP2CAs are not involved in negatively regulating ABA signalling during germination (Bhaskara et al., 2012). This aligns with the low preponderance of HAI PP2CAs in dry seeds. Yet, HAI2 (also known as HONSU), the only HAI present in dry seeds, has been proposed to play a role in seed dormancy (Kim et al., 2013). The *snrk2.6* mutant does not show an altered germination phenotype, but the double mutant *snrk2.2 2.3* of both seed class III SnRK2s displays a strongly reduced ABA sensitivity, though mutations of all class III SnRK2s are required to obtain an ABA-blind phenotype during germination (Fujii et al., 2007; Fujii and Zhu, 2009; Nakashima et al., 2009).

In summary, proteomic data show that the seed ABA core signalosome composition is characterized by (i) the presence of all ABA receptor types with a dominance of group III, (ii) the overrepresentation of AHG1 within the seed PP2CA pool, and (iii) the presence of only two ABA-responsive SnRK2 kinases. Importantly, genetic studies match with the seed ABA signalosome composition, illustrating the value of quantitative proteomic data to explore the functioning of ABA signalling during seed imbibition.

### A signalosome shaped to handle fluctuating ABA levels

The absolute concentration of ABA in dry seeds poorly correlates with their germinative capacity. ABA levels rapidly decline during seed imbibition and stay low in germinating seeds but reaccumulate in non-sprouted seeds (i.e. dormant seeds or seeds imbibed under stress conditions) (Ali-Rachedi et al., 2004; Toh et al., 2008). Studies using inhibitors to prevent *de novo* synthesis strengthen the concept that fluctuations in ABA levels during imbibition determine seed fate (Debeaujon and Koornneef, 2000; Ali-Rachedi et al., 2004; Née et al., 2017a). Tacitly, this indicates that the molecular systems surveying cellular ABA concentrations along seed imbibition to initiate ABA signalling are critical for the control of germination.

The three different groups of ABA receptors are characterized by distinct biochemical features. ABA globally enhances the inhibitory activity of all receptors towards PP2CAs, but only group III receptors strictly depend on ABA to execute their role. In contrast, group I receptors and to a lesser extent members of group II can inhibit canonical PP2CAs in the absence of ABA or at resting ABA levels. Group III receptors are specific to angiosperms. In contrast to other RCARs, group III RCARs form homodimers in solution, making them unable to interact with PP2CAs. Upon ABA binding, group III monomerization enables interaction and regulation of PP2CA activity; hence, in the absence of or at low levels of ABA, these RCARs likely accumulate in latent dimeric forms (Rodriguez et al., 2019; Sun et al., 2019; Sun et al., 2020). Initial *in vitro* biochemical studies first pointed out differences between receptors regarding their efficiency in inhibiting PP2CA (Antoni et al., 2012). More recently, two complete combinatorial analyses investigated the 126 possible Arabidopsis RCAR-PP2CA pairings using *in vivo* reconstituted systems and provided quantitative data to better understand the wiring of the initial step of ABA signal transduction (Tischer et al., 2017; Ruschhaupt et al., 2019). These studies clearly demonstrated that although most of the possible RCAR-PP2CA combinations appeared functional in terms of inhibiting PP2CA, the efficiency, or the requirement of ABA to relieve the PP2CA-mediated shutdown of ABA responses, strongly differed between RCARs.

**Figure 2:**
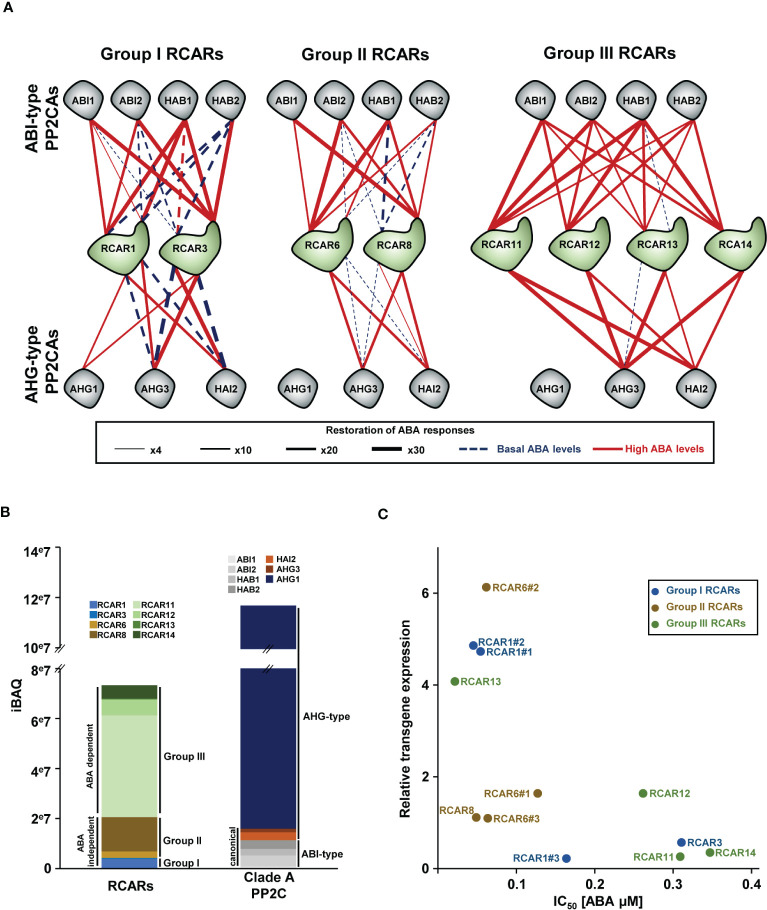
Initiation of ABA signalling at basal and high hormone levels through receptor-phosphatase wirings. **(A)** RCAR ability to repress PP2CAs *in vivo.* Connections at basal or high (10 µM exogenously applied) ABA concentrations are represented by dashed and full lines, respectively. Line thickness is related to the strength of the inhibition. The schematic representation was built based on the data from the study by Tischer et al., 2017. Experiments were performed in a reconstituted system in protoplast overexpressing *RCARs* and *PP2CAs* together with a reporter construct (*pRD29B::LUC*) as ABA response readout. **(B)** Direct comparison of RCAR and PP2CA abundances in dry seed. Intensity-based absolute quantification (iBAQ) values for the two initial components of the ABA core signalling pathway were obtained from the study by Mergner et al., 2020, and presented as a histogram of cumulated abundances. **(C)** Capacity of different RCARs to trigger ABA-mediated germination arrest in function of their overexpression level. RNA expression data and [ABA] IC_50_ were obtained from the study by Yang et al., 2016, and plotted against each other. The presence of # indicates independent overexpression lines. For reference, the [ABA] IC_50_ of the wild type seeds in this assay is around 0.6 µM.

We exploited these data to map the initial information flux and its requirement for ABA in seeds (**Figure 2A**). Strikingly, it emerges that AHG1 is not inhibited by group III and group II RCARs; only group I receptors (RCAR1 and RCAR3) were able to mildly inhibit AHG1 in the presence of high ABA levels. Another interesting characteristic of the seed RCAR-PP2CA signalling network is that RCAR1 and RCAR3 strongly repress canonical PP2CAs even at low ABA levels. Inhibition of HAI2 or AHG3 is independent of the ABA concentration, but high ABA levels enhance their ability to repress the ABI-type PP2CAs. Seed group II receptors (RCAR8 and RCAR6) mildly inhibit canonical PP2CAs at basal ABA levels, but higher concentrations strongly increase their efficiency. Activity of group III RCARs requires high ABA levels but, in turn, provides an efficient and pan-specific inhibition system for canonical PP2CAs.

The inhibitory complex RCAR-PP2CA is formed in a 1:1 ratio (Soon et al., 2012), and the quantitative proteomic data from the Arabidopsis Atlas Proteome allow for roughly addressing the relative receptor-phosphatase stoichiometry in dry Arabidopsis seeds (Mergner et al., 2020). In this organ, the AHG1 protein level is generally greater than the ABA receptor pool. However, when excluding AHG1, ABA receptors exceed the total PP2CA abundance by approximately four times (**Figure 2B**). Cumulative protein levels for the ABA-independent group I and II receptors are roughly comparable to the total canonical PP2CA abundance. This suggests that in seeds, a non-negligible fraction of the canonical PP2CA pool is possibly *de facto* under some inhibitory constraint independent of the ABA level. Moreover, considering the high ABA affinity of group I and II receptors (K_d_ approx. 1 µM) and the high abundance of RCAR8 in seeds, these receptors could perform much of the PP2CA inhibition until the ABA concentration drops to a minimal level (i.e. in germinating seeds). The strictly ABA-dependent group III RCARs possess a low intrinsic affinity for ABA (K_d_ > 50 µM) in the absence of PP2CAs, but a high phosphatase-to-receptor ratio was shown to increase their binding affinity (Dupeux et al., 2011; Moreno-Alvero et al., 2017; Tischer et al., 2017). Yet, group III receptors represent more than two-thirds of the total receptor pool in dry seeds, and their cumulative abundance is approximately three times the canonical PP2CA pool size (**Figure 2B**). This indicates that group III receptors require high cellular ABA levels for activation, but, in turn, their high abundance may provide the powerful system required to fully shut off canonical PP2CAs in seeds.

Further insights about the respective roles of different RCARs in the context of different ABA concentrations can be gained from the analysis of genetic data. A study comparing the effect of overexpression of each RCAR in an ABA-mediated germination arrest assay revealed that some receptors were more effective than others during seed imbibition (Yang et al., 2016). We took advantage of this quantitative data to address the relation between ABA levels and the activation of different seed RCARs by examining the ABA concentration required to trigger germination arrest (IC_50_) in relation to the relative level of *RCAR* overexpression. Overexpression of any receptor increased ABA sensitivity during seed imbibition. But seed group II receptors appeared to repress germination at lower ABA concentrations compared to group III RCARs (**Figure 2C**). Besides ABA sensitivity during germination, it has been shown that overexpression of *RCAR3* increased seed dormancy *sensu stricto* (Saavedra et al., 2010). Another study suggested that overexpression of WT group I *RCARs* does not obviously modify dormancy, but overexpression of an *RCAR4* neo-morphic isoform, in which three positions located at the PP2CA interaction interface were exchanged with the corresponding RCAR1 sequence, increased dormancy (Tischer et al., 2017). Although further investigations are required to clearly address the role of group I RCARs in seed dormancy, the existing data indicate that they possibly have a role in securing some amount of positive ABA signalling to prevent the resumption of growth in dormant seeds, despite the dropping ABA levels during early imbibition. The contribution of the different RCARs in the response at low and high ABA levels can also be deduced from the analysis of single to multiple *rcar* mutants. Compared to the WT seed, germination of *rcar6* single and *rcar3 8 10* triple mutants (noting that RCAR10 is not accumulated in seed) are less sensitive to low ABA concentrations (<1 µM) but are fully inhibited at higher doses (>5 µM). Despite being the most abundant receptor in seeds, the *rcar11* mutant shows a WT-like ABA sensitivity during germination, but the quadruple *rcar10 11 12 14* mutant, in which all the seed-abundant group III receptors are not functional, can germinate in presence of 5-10 µM ABA (Park et al., 2009; Gonzalez-Guzman et al., 2012). These observations support a role of group I and II receptors in initiating signalling at low ABA levels and the requirement of group III activation to secure germination arrest at higher ABA concentrations. Yet, ABA-blind phenotypes during germination are achieved only in seeds combining loss-of-function mutations for the three receptor groups, such as the quintuple *rcar3 8 10 11 14* or sextuple *rcar3 8 10 11 12 14* mutants. Interestingly, only the sextuple mutant including a loss-of-function allele for the second most abundant group III (RCAR12) receptor in seeds is insensitive to ABA during cotyledon greening (Gonzalez-Guzman et al., 2012). This further points at an essential role of group III RCARs in late and post-germination events after the eventual (re)activation of ABA synthesis.

Altogether, biochemical and genetic data suggest that in seeds, group I and II RCARs may function to avoid the abrupt shutdown of responses in early imbibitional phases during the initial drop of ABA concentration. In addition, as these receptors are strongly activated at low ABA concentrations, subtle increases from the resting level may provide strong responses and, thus, a rapid and cost-effective way to delay germination in presence of low-intensity or short-term stress conditions. In contrast, the overrepresentation of group III receptors likely reflects the need for an amplified response to secure germination arrest at later stages of imbibition when ABA levels are increasing in non-germinated seeds. The requirement of group III RCAR activity for a leakproof inhibition of canonical PP2CAs in seeds is fully coherent with the necessity of ABA *de novo* synthesis for germination arrest.

### Genuine and promiscuous relations of clade A PP2C family

Molecular and genetic data suggest overlapping but also distinct functions within the clade A PP2Cs. Redundancy within the ABI-type PP2CAs is illustrated by the requirement of combinatorial null mutations to obtain clear ABA-hypersensitive phenotypes and the observation that the same hypermorphic G to D mutation in *abi1-1* or *abi2-1* alleles leads to ABA insensitivity in germination assays (Koornneef et al., 1984; Rubio et al., 2009). In contrast, *AHG1* and *AHG3* genes have been identified through a genetic screen in which the loss-of-function of one gene leads to an ABA-hypersensitive germination phenotype (Yoshida et al., 2006; Nishimura et al., 2007). However, the double *ahg1 3* mutant displays constitutive ABA responses during imbibition, as its seeds display extreme dormancy and ABA-hypersensitivity phenotypes, indicating that *AHG1* and *AHG3* are partly genetically redundant (Née et al., 2017a). Combining *ahg3* with *abi1*, or *hab1* loss-of-function alleles enhances ABA hypersensitivity during germination, indicating partial genetic redundancy of AHG3 with the ABI-type PP2CAs (Rubio et al., 2009). Although the phenotype of the respective mutants was described years ago, it is not yet clear whether genetic redundancy of PP2CAs is reflected at the molecular level by an overlap of downstream targets and/or the control of genetically additive but distinct molecular pathways. The presence of noticeable to extreme germination phenotypes for some single or low-order *pp2ca* mutants suggests that not all PP2CAs control ABA responses via the same *modus operandi*. The nine Arabidopsis clade A PP2Cs are localized in the nucleus, but, in contrast to AHG-type PP2CAs, ABI-type PP2CAs are also found in the cytosol, further supporting the existence of separated cellular functions (Nishimura et al., 2018).

**Figure 3:**
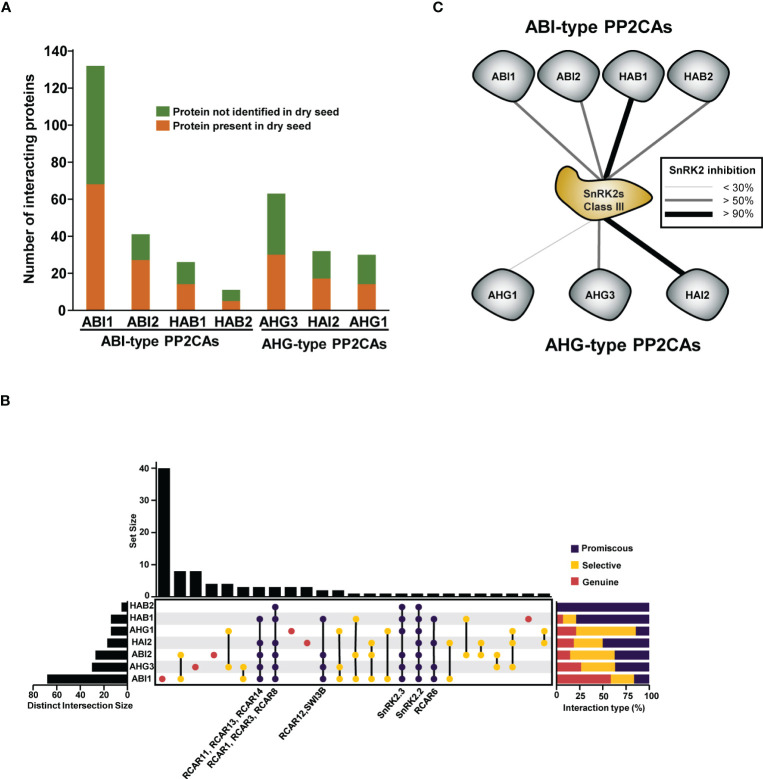
Network connectivity of PP2CAs present in seeds. **(A)** Bar graph representing the numbers of non-redundant protein-protein interactions for each PP2CA present in seeds. PP2CA interacting proteins were further filtered for their presence in dry seed according to the study by Mergner et al., 2020. **(B)** Overlaps of protein interaction between the different PP2CAs present in seed. Left graph shows the number of interacting proteins accumulated in seeds for each PP2CA. The centred plot shows network connection as nodes representing unique or shared interactions, and the upper graph the number of proteins in each node. Interactions were considered as selective or promiscuous when shared with less or more than half of the seed PP2CA pool and are shown in orange and red, respectively. PP2CA-unique sets of interaction are shown in green. The content of promiscuous sets of protein interaction is labelled. Right graph summarizes the relative level of promiscuity for each PP2CA. **(C)** Ability of PP2CAs to inhibit class III SnRK2s *in vivo*. Connection line thickness represents the strength of the inhibition. The schematic representation was built based on the data from the study by Ruschhaupt et al., 2019. Experimentations were performed using a reconstituted system in a yeast strain harbouring a stable genomic integration of a *4xABRE* and a minimal promoter to control luciferase expression. The different *PP2CA* were expressed together with *SnRK2.6* and *ABF2* and the LUC activity used as ABA response read out.

Protein interaction databases are powerful resources that can be used to investigate common or specific protein roles. The Transcriptionally Regulated ABA Interactome Network (TRAIN), which is an ABA-focused interactome containing 500 paired interactions, and the phytohormone interactome (PhI) network, which is a systematic (unbiased design) high-throughput yeast interaction assay that contains 2,072 interaction pairs, have strongly contributed to filling (in a publicly available way) protein interaction databases with PP2CA-interacting proteins (Lumba et al., 2014; Altmann et al., 2020). We retrieved protein interactions for the seven seed PP2CAs from the Biogrid and IntAct repositories. We found a total of 190 non-redundant interactors, but, as many of these physical interactions were evidenced in reconstituted systems and not in the native context, we further filtered interactors for their occurrence in dry seeds. According to the Arabidopsis Atlas Proteome, approximately half of the currently known PP2CA-interacting proteins are present in seeds (**Figure 3A**). Using this subset of proteins, we attempted to address network connectivity between isoforms by analysing their interaction overlaps (**Figure 3B**). We found that the different PP2CAs engage in promiscuous interactions but also selective or genuine interactions. At the exception of HAB2, all seed PP2CAs have unique interaction partners, but ABI1 showed the highest apparent rate of genuine interactions. Because ABI1 was more frequently investigated as a prototypical PP2CA, the number of referenced protein interactions for ABI1 is higher than for other members. Similarly, the low number of interactions evidenced for HAB2 possibly overstates the apparent discrepancies regarding ABI1 and HAB2 genuine interactions. Still, unique sets of partners are also present for PP2CAs with a similar number of referenced interactions. Interestingly, the apparent rate of genuine interactions for AHG1 and AHG3 is higher than 20% (**Figure 3B**); this aligns with the clear phenotype of their single mutants. The proportion of selective interactions (shared between some PP2CAs) is important within PP2CAs networks, but the strongest linkages are observed between ABI1-ABI2 and AHG1-AHG3. These molecular interconnections possibly reflect redundant functions in agreement with the enhancement of ABA hypersensitivity in the corresponding double mutants. Expectedly, the two other components of the core signalosome (ABA receptors and class III SnRK2s) appear to have the widest interconnections (shared interactions between most PP2CAs). Likely, promiscuous interactions with the SnRK2 class III hub explains a big part of the genetic redundancy between PP2CAs. Nevertheless, a recent study analysing the capacity of the nine Arabidopsis PP2CAs to weaken SnRK2-transduced transcriptional ABA responses in an ABA signalling cascade rebuilt in yeast demonstrated differential efficiencies between PP2CAs regarding kinase repression (Ruschhaupt et al., 2019). Although the assays were conducted with SnRK2.6 (a class III representative), which was not detected as protein in dry seeds, this study highlighted that ABI-type PP2CAs, HAI2, and AHG3 repress class III SnRK2-mediated signalling more efficiently than AHG1, which was the weakest repressor in this assay (**Figure 3C**).

Altogether, the analysis of biochemical, interaction, and genetic data for the different seed PP2CAs suggests that although all members are negative regulators of ABA responses, single PP2CAs may promote seed sprouting via common and unique mechanisms, providing robustness (deactivation of SnRK2s) and flexibility (negative regulation of ABA responses via genuine pathways) during the progression of germination. Possibly, distinct signals (i.e. seed age, stressor A or B) may be integrated at the level of different PP2CAs to adjust seed germination characteristics with external conditions.

### The unresolved case of AHG1

The expression pattern of *AHG1* differs substantially from other canonical *PP2CAs*, as its mRNA is exclusively detected in late maturing or dry seed but not during the vegetative growth phase, not even after ABA treatment (Nishimura et al., 2007). The presence of AHG1 is therefore a unique feature of the seed ABA signalosome. As such, *ahg1* mutants or plants ectopically overexpressing *AHG1* in leaves do not show typical ABA-related stress phenotypes such as increased transpiration after seedling establishment (Nishimura et al., 2007; Née et al., 2017a; Nishimura et al., 2018). This suggests that the role of AHG1 is limited to controlling germination. Notably, AHG1 is not regulated by group II and III and only poorly inhibited by group I RCARs even at high ABA concentrations (Tischer et al., 2017; Ruschhaupt et al., 2019) (**Figure 2A**).

**Figure 4:**
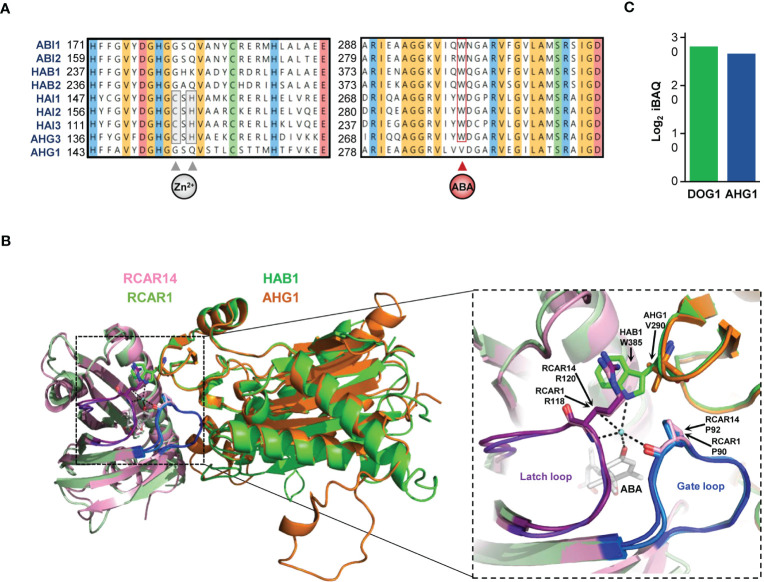
Unorthodox features of AHG1 primary amino acids sequence. **(A)** Conserved regions of Arabidopsis clade A proteins. Amino acid positions are labelled on the left. The cysteine and histidine residues implied in the coordination of the zinc atom in the zinc finger domain and the tryptophan lock residue implied stabilization of the ABA-RCAR-PP2CA tertiary complexes are marked by arrows, and their degree of conservation is boxed. Shading indicates strict conservation among all 9 PP2CAs and represents amino acid biochemical classes: yellow = aromatic, red = acidic, blue = basic, orange = aliphatic, green = neutral. **(B)** Three-dimensional representation of the ABA-RCAR-PP2CA tertiary complex. X-ray crystallographic structures of Arabidopsis ABA-bound RCAR14 superimposed to the ABA-bound RCAR1 monomer in complex with HAB1. Structural modelling of AHG1 is superimposed to HAB1 subunit in complex with RCAR14. Ribbons of RCAR14 are shown in pink, RCAR1 in dark green, HAB1 in bright green, and AHG1 (AA 138-416) in orange. The right panel shows an enlarged view of the squared region focusing on the water-mediated bonding between the ABA molecule, the gate/latch loops of RCARs, and the tryptophan lock of canonical PP2CAs. The ABA molecule and the residue engaging in hydrogen bonding (dashes) with the water molecule (blue sphere) are shown as sticks. The latch and gate loops are shown in purple and blue, respectively. **(C)** Direct comparison of DOG1 and AHG1 protein abundances in dry seed. Log_2_ intensity-based absolute quantification (iBAQ) values for were obtained from the study by Mergner et al., 2020, and presented as a histogram.

All RCARs share two conserved structural domains: the “gate” and the “latch” loops surrounding the ABA binding pocket. For RCARs to interact with PP2CAs, these loops must change from an open to a closed conformation. Importantly, the RCAR-PP2CA tertiary inhibitory complex is stabilized by PP2CA’s “tryptophan lock” residue (Melcher et al., 2009); this residue is a unique feature of the clade A phosphatases, strictly conserved in 8 Arabidopsis PP2CAs, but it is replaced by a valine in AHG1 (**Figure 4A**). In canonical PP2CAs, the amine group in the indole ring of the tryptophan side chain is connected through water-mediated hydrogen bonding to RCARs’ gate/latch loops and the ABA molecule (**Figure 4B**). However, in AHG1, the valine side chain is aliphatic and less bulky than tryptophan, so AHG1-RCAR complexes may be labile. This is further supported by the experimental observation that mutation of the tryptophan lock to alanine in canonical PP2CAs results in the loss of RCAR-mediated inhibition (Dupeux et al., 2011). Given this divergence at a critical amino acid position and the high AHG1 levels in seeds which exceeds the pool of group I ABA receptors by 20 times, it can be hypothesized that RCARs are scarcely involved in AHG1 activity regulation during germination. In addition to RCAR unresponsiveness, initial *in vitro* biochemical assays have indicated that deactivation of OST1/SnRK2.6 by AHG1 is less efficient than its deactivation by other canonical PP2CAs, even after prolonged incubation times (Umezawa et al., 2009; Antoni et al., 2012). This hypothesis is further strengthened by the aforementioned analysis of the PP2CA-SnRK2s hub in a reconstituted system *in vivo* (**Figure 3C**) (Ruschhaupt et al., 2019).

The structural mechanism of canonical PP2CAs’ inhibition by RCARs is based on a molecular mimicry of PP2CA-SnRK2 interaction interfaces. In the tertiary complex, the latch loop of the ABA receptor invades the PP2CA catalytic site in a similar fashion as the target substrate: the SnRK2 activation loop. Reciprocally, the clade A phosphatase’s tryptophan lock inserts into the SnRK2 catalytic cleft (Soon et al., 2012). Thereby, this tryptophan residue participates in kinase docking and fully inhibits the kinase activity after activation loop dephosphorylation. In AHG1, the absence of this structural determinant possibly prevents the complete shutoff of the ABA core signalling kinases.

Nevertheless, so far, the RCAR-AHG1-SnRK2 relay has only been investigated outside of the native seed context. Possibly, additional factors present in seeds either supplant or bridge this atypical PP2CA with the other ABA core signalosome components. Interestingly, the protein of unknown function DELAY OF GERMINATION 1 (DOG1), a master regulator of seed dormancy, physically interacts with AHG1 in seed tissue (Née et al., 2017a). The role of DOG1 in controlling dormancy has been evidenced through natural variation studies in Arabidopsis, pointing out its crucial role for plant adaptation (Alonso-Blanco et al., 2003). The completely non-dormant phenotype of *dog1* demonstrates that this regulator is required for this seed-specific trait (Bentsink et al., 2006; Née et al., 2017a). In contrast to absolute ABA concentration, protein levels of DOG1 in dry seed correlate with the deepness of dormancy (Nakabayashi et al., 2012); further, DOG1 protein levels are high and comparable to AHG1 in dry seed (**Figure 4C**). DOG1 also interacts with AHG3 in seed and with HAIs in yeast cells but not with ABI-type PP2CAs (Née et al., 2017a; Nishimura et al., 2018). The central role of DOG1 in dormancy control as well as the existence of DOG1-PP2CA protein complexes (the so-called DOG1-PP2C module) have also been shown for other species isoforms (Ashikawa et al., 2010; Née et al., 2015; Huo et al., 2016; Wang et al., 2020; Yu et al., 2020).

Genetic analyses carried out by two independent groups indicated that DOG1 controls seed dormancy via negative regulation of AHG1 and AHG3 function. The extremely dormant phenotype of the *ahg1 3* double mutant is epistatic to the totally non-dormant phenotype of *dog1* seeds (Née et al., 2017a). Co-overexpression of *DOG1* reverts the ABA insensitivity of seeds overexpressing *AHG1* (Nishimura et al., 2018). Although neither of the two studies could determine that DOG1 inhibited AHG1 or AHG3 phosphatase activity *in vitro* using the standard RRA(pT)VA phosphopeptide as substrate, in the study by Nishimura and co-authors, who used a phosphopeptide corresponding to the sequence of the SnRK2.6 activation loop, the authors reported a ~40% inhibition of AHG1 by a threefold excess of DOG1 *in vitro*. Hence, these authors stated that DOG1 controls the activation status of class III SnRK2s by directly inhibiting AHG1 catalytic activity. Taking into account the large AHG1 pool size in seeds, a reduction by half of its phosphatase activity *per se* is unlikely to allow an efficient activation of ABA responses. This suggests that additional unknown factors, not included in *in vitro* assays, are required to produce DOG1’s inhibitory effect on phosphatase activity, or/and that *in vivo* DOG1 also impedes AHG1 functions independently of AHG1 catalytic properties.

Lastly, crystallographic studies have revealed the presence of a CCH zinc finger (ZF) domain in AHG-type PP2CAs, which in AHG3 involves Cys146, His148, and Cys208/210. This domain is close to the phosphatase-receptor interaction interface, and a Cys ligand point mutation severely impairs AHG3 catalytic activity, suggesting that the ZF is important for structural integrity. The Cys208/210 residues of AHG3 are strictly conserved in all AHG-type PP2CAs, but Cys146 and His148 are absent in AHG1, further distinguishing this phosphatase from its closest relatives (**Figure 4A**).

In brief, structural, molecular, and genetic data commonly point to AHG1 as an unorthodox PP2CA.

### Rewiring information fluxes within the core pathway

Seed water uptake allows for a rapid resumption of cellular metabolism, permitting a quick activation of molecular signalling (Paszkiewicz et al., 2017; Nietzel et al., 2020), whereas the quiescent state of dry seeds prevents translational or proteasomal cellular activities (Bewley et al., 2013). Thus, the ABA core signalosome protein equipment stored in dry seeds is defined by transcriptional and translational activity during seed maturation. Its configuration is a major determinant of germination characteristics upon imbibition, as this backbone integrates internal and external signals to mediate germination progression. Yet, forthcoming imbibitional conditions cannot be fully predicted during seed maturation, notably after long-distance dispersal or storage time in the soil seed bank (SSB). Hence, timely germination requires permanent readjustment of the ABA pathway operations according to external cues. Several reviews have emphasized the prominent role of proteome changes during seed imbibition through selective translation of mRNA, targeted protein degradation, and post-translational modifications (PTMs) in the control of germination (Arc et al., 2011; Oracz and Stawska, 2016; Née et al., 2017b).

**Figure 5:**
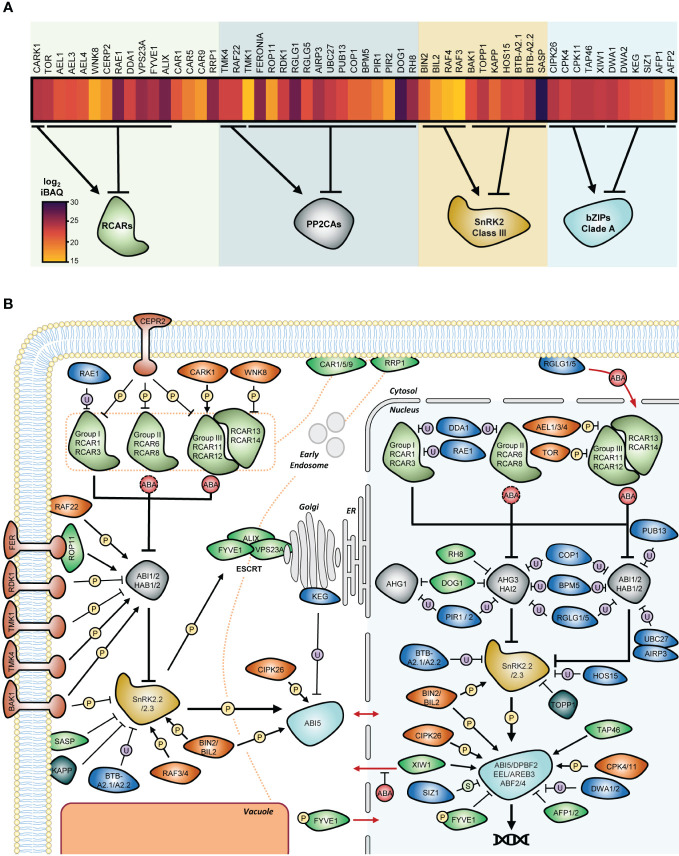
Molecular systems controlling the operation of ABA core signalosome present in dry seed. **(A)** Relative abundances of proteins affecting the performance of ABA signalosome in dry seed of *Arabidopsis thaliana*. Log_2_ transformed intensity-based absolute quantification (iBAQ) values were obtained from the study by Mergner et al., 2020, and presented as a heat map where dark tones indicate high accumulation. The effect on target core components is depicted below the heat map. **(B)** Extended model of ABA signalling in dry seed. Information fluxes within the core pathway components and the pathway’s first shell of regulation is shown as thick and thin lines, respectively. Proteins involved in the UPS-mediated degradation pathways are shown in blue, kinases in orange, phosphatases in grey, and proteins with other or unknown molecular functions in green. The strict or moderate requirement of high ABA level for functionality is depicted as red full or dashed circles, respectively. Protein movement between cytosol and nucleus is indicated by red arrows. The trafficking and removal of ABA receptors by the endocytic pathways in the cytosol is depicted by orange dashed lines. Full names of proteins involved in the ABA signalosome are presented in the main text. For clarity reasons, the specificity of regulation at the levels of nuclear PP2CAs and bZIP transcription factors was not fully depicted; see main text for information. P, phosphorylation; U, ubiquitination; S, SUMOylation; ER, Endoplasmic Reticulum.

Recent research on ABA signalling has led to the identification of several proteins acting as biological rheostats to terminate or enhance ABA responses by modulating stoichiometry and/or activities of core signalosome components. These switches leading to changes in ABA signal fluxes are controlled by PTMs or protein interactions in an ABA-dependent or -independent fashion. We screened the scientific literature and found 80 proteins involved in the direct regulation of ABA core signalling (**Supplementary Table S2**). Next, we filtered the entries to retain 55 proteins that were experimentally detected as present in dry seeds according to the Arabidopsis Atlas Proteome (**Table 1** and **Figure 5A**).

**Table 1:** Peripheral regulators of the core ABA signalling pathway present in dry seed.

Gene ID	Annotation	Protein class	Localization	Impact on ABA response	Germination phenotype	Reference(s)
*AT3G17410*	CARK1	Receptor-like cytoplasmic kinase (RLK)	Cytosol	Positive	Reduced ABA sensitivity in *cark1* mutant	(Zhang et al., 2018; Li et al., 2019a)
*AT5G11850* *AT1G18160*	M3Kδ1/ RAF3 M3Kδ7/ RAF4	Mitogen Activated Protein kinase kinase kinase (MAPKKK) B3	Cytoplasm	Positive	Reduced ABA sensitivity in *the m3kδ1 m3kδ6-1 m3kδ7* mutant	(Takahashi et al., 2020)
*AT2G24360*	RAF22	Mitogen Activated Protein kinase kinase kinase (MAPKKK) C6	Plasma Membrane	Negative	Not shown	(Sun et al., 2022)
*AT4G18710* *AT1G06390*	BIN2 BIL2	Glycogen synthase kinase 3 (GSK3)-like kinase	Cytoplasm, nucleus	Positive	Reduced ABA sensitivity *in bin2-3 bil1 bil2* mutant	(Cai et al., 2014)
*AT4G09570*	CPK4	Calcium-dependent protein kinase	Cytoplasm, nucleus	Positive	Reduced ABA sensitivity in *cpk4* mutant	(Zhu et al., 2007)
*AT1G35670*	CPK11	Calcium-dependent protein kinase	Cytoplasm, nucleus	Positive	Reduced ABA sensitivity in *cpk11* mutant	(Zhu et al., 2007)
*AT1G50030*	TOR	Serine/threonine-protein kinase	Nucleus	Negative	Enhanced ABA sensitivity in *raptor* mutant (TOR kinase complex subunit)	(Wang et al., 2018)
*AT5G41990*	WNK8	With no lysine (K) (WNK) kinase	Cytoplasm, nucleus	Negative	Enhanced ABA sensitivity in *wnk8* mutant	(Waadt et al., 2019)
*AT5G21326*	CIPK26	Ca2+ regulated serine-threonine protein kinase	Cytoplasm	Positive	Enhanced ABA sensitivity in overexpression line	(Lyzenga et al., 2013)
*AT2G25760* *AT3G03940* *AT5G18190*	AEL1 AEL3 AEL4	Casein Kinase	Nucleus	Negative	Enhanced ABA sensitivity in double and triple *ael* mutants	(Chen et al., 2018)
*AT1G72180*	CEPR2	Receptor-like protein kinase (RLK)	Plasma membrane	Negative	Not shown but redundant with *pxy pxl2* in seedling establishment	(Yu et al., 2019)
*AT4G33430*	BAK1	Receptor-like protein kinase (RLK)	Plasma membrane	Negative	Enhanced ABA sensitivity of *bak1* mutant	(Deng et al., 2022)
*AT1G66150*	TMK1	Receptor-like protein kinase (RLK)	Plasma membrane	Positive	Reduced ABA sensitivity in *tmk1* mutant	(Li et al., 2021; Yang et al., 2021)
*AT3G23750 *	TMK4	Receptor-like protein kinase (RLK)	Plasma membrane	Negative	Enhanced ABA sensitivity in *tmk4* mutant	(Li et al., 2021)
*AT3G51550*	FER	Receptor-like protein kinase (RLK)	Plasma membrane	Negative	Enhanced ABA sensitivity of *fer* in seedling establishment	(Yu et al., 2012; Chen et al., 2018)
*AT4G34220*	RDK1	Receptor-like protein kinase (RLK)	Plasma membrane	Positive	Reduced ABA sensitivity of *rdk1* mutant	(Kumar et al., 2017)
*AT5G19280*	KAPP	Protein phosphatase 2C	Plasma membrane	Negative	Enhanced ABA sensitivity of *kapp* mutant	(Manabe et al., 2008; Lu et al., 2020)
*AT2G29400*	TOPP1	Phosphoprotein phosphatase type PP1	Nucleus	Negative	Enhanced ABA sensitivity in *topp1* mutant	(Hou et al., 2016)
*AT5G62880*	ROP11	Rho GTPase	Plasma membrane	Negative	Enhanced ABA sensitivity in *rop11 arac10-3* mutant	(Yu et al., 2012; Chen et al., 2018)
*AT1G20110*	FYVE1	ENDOSOMAL SORTING COMPLEX REQUIRED FOR TRANSPORT complex protein	Endosomes, nucleus	Negative	Enhanced ABA sensitivity in *fyve1* mutant	(Belda-Palazon et al., 2016; Li et al., 2019a)
*AT3G12400*	VPS23A	ENDOSOMAL SORTING COMPLEX REQUIRED FOR TRANSPORT complex protein	Endosomes	Negative	Enhanced ABA sensitivity in *vps23a* mutant	(Yu et al., 2016)
*AT1G15130*	ALIX	ENDOSOMAL SORTING COMPLEX REQUIRED FOR TRANSPORT complex protein	Multivesicular bodies, endosomes	Negative	Enhanced ABA sensitivity in *alix* mutant	(García-León et al., 2019)
*AT5G37740 AT1G48590* *AT1G70790*	CAR1 CAR5 CAR9	Calcium-dependent lipid-binding proteins	Nucleus, plasma membrane	Positive	Reduced ABA sensitivity in triple *car1 car5 car9* mutant	(Rodriguez et al., 2014)
*AT1G15470*	XIW1	Transducin/WD40 superfamily protein	Cytosol, nucleus	Positive	Reduced ABA sensitivity in *xiw1* mutant	(Xu et al., 2019)
*AT5G45830*	DOG1	DOG1-like protein family	Nucleus	Positive	Reduced ABA sensitivity in *dog1* mutant	(Bentsink et al., 2006; Née et al., 2017a)
*AT4G00660*	RH8	DEAD Box RNA Helicase	Nucleus, P-body	Positive	Reduced ABA sensitivity in *rh8* mutant	(Baek et al, 2018)
*AT5G53000*	TAP46	2A phosphatase associated protein	Cytosol, nucleus	Positive	Reduced ABA sensitivity in *tap46* mutant	(Hu et al., 2014)
*AT3G14067*	SASP	Subtilase	Plasma membrane	Negative	Enhanced ABA sensitivity in *sasp* mutant	(Wang et al., 2018)
*AT3G48860*	RRP1	Clathrin-related protein	Plasma membrane	Positive	Reduced ABA sensitivity in *scd2* mutant	(Hou et al., 2020)
*AT1G69260*	AFP1	ABI-five binding protein	Nucleus	Negative	Enhanced ABA sensitivity in *afp1* mutant, extreme insensitivity in overexpression line	(Lopez-Molina et al., 2003; Garcia et al., 2008; Lynch et al., 2017)
*AT1G13740*	AFP2	ABI-five binding protein	Nucleus	Negative	Enhanced ABA sensitivity in *afp2* mutant, extreme insensitivity in overexpression line	(Garcia et al., 2008; Lynch et al., 2017)
*AT5G60410*	SIZ1	SUMO E3 ligase	Nucleus	Negative	Enhanced ABA sensitivity in *siz1* mutant	(Miura et al., 2009)
*AT2G19430* *AT1G76260*	DWA1 DWA2	DDB1-binding WD40 proteins	Nucleus	Negative	Enhanced sensitivity in *dwa1 dwa* mutant	(Lee et al., 2010)
*AT5G67320*	HOS15	CUL4-DDB1-based E3 ubiquitin ligase	Nucleus	Negative	Enhanced ABA sensitivity in *hos15* mutant	(Ali et al., 2019)
*AT2G35330*	PIR1	RING finger E3 ubiquitin ligase	Nucleus	Positive	Reduced ABA sensitivity in *pir1* mutant	(Baek et al., 2019)
*AT1G32530*	PIR2	RING finger E3 ubiquitin ligase	Nucleus	Positive	No difference in *pir2* mutant; enhanced ABA sensitivity in overexpression line	(Baek et al., 2019)
*AT3G01650 AT1G67800*	RGLG1 RGLG5	RING finger E3 ubiquitin ligases	Plasma membrane, nucleus	Positive	Reduced ABA sensitivity in *rglg1 rglg5* mutant	(Wu et al., 2016; Belda-Palazon et al., 2019)
*AT2G32950*	COP1	RING finger E3 ligase ligase	Cytoplasm, nucleus	Positive	Reduced ABA sensitivity in *cop1* mutant in darkness	(Chen et al., 2020)
*AT5G21010*	BPM5	BTB/POZ AND MATH DOMAIN protein	Nucleus	Positive	Enhanced ABA sensitivity in overexpression line	(Julian et al., 2019)
*AT5G41330* *AT3G09030*	BTB-A2.1 BTB-A2.2	BTB domain proteins	Cytoplasm, nucleus	Negative	Enhanced ABA sensitivity in double and triple *btb-a2.1 2 3* mutants	(Cai et al., 2020)
*AT5G50870*	UBC27	Ubiquitin-conjugating enzyme	Cytoplasm, nucleus	Positive	No change in mutant	(Pan et al., 2020)
*AT3G09770*	AIRP3	RING finger E3 ubiquitin ligase	Plasma membrane	Positive	Reduced ABA sensitivity in *airp3* mutant	(Pan et al., 2020)
*AT5G41560*	DDA1	Substrate receptor protein for CRL4-CDD complex	Nucleus	Negative	Reduced ABA sensitivity in overexpression line	(Irigoyen et al., 2014)
*AT1G80670*	RAE1	F-box protein component of SCF-type E3 ligase complex	Cytoplasm, nucleus	Negative	Enhanced ABA sensitivity in RNAi-lines, reduced ABA sensitivity in overexpression line	(Li et al., 2018)
*AT3G46510*	PUB13	Plant U-box (PUB) E3 ligase	Cytoplasm, nucleus	Positive	Reduced ABA sensitivity in *pub13* mutant	(Kong et al., 2015)
*AT5G13530*	KEG	RING finger E3 ubiquitin ligase	Trans-Golgi	Negative	Reduced ABA sensitivity in overexpression line	(Stone et al., 2006; Gu and Innes, 2011; Liu and Stone, 2013)

Proteins are listed with corresponding AGI code, protein class and abbreviation used in the mentioned references (for full name see main text). Subcellular localization data, ABA and seed germination related phenotypes were experimentally observed.

In the following sections, we summarize the role of these peripheral factors in the direct regulation of ABA core signalosome components. Finally, we incorporated these molecular systems into the core pathway to propose an extended model illustrating the flexibility of ABA signalling in seeds regarding the nature and amplitude of the responses (**Figure 5B**).

### (De-)Sensitizing ABA hormone perception

The cumulative protein pool size of group I and II RCARs that can mediate some extent of inhibitory activity at basal ABA levels is greater than the pool of canonical PP2CAs (**Figure 2B**). Implicitly, this indicates that consistent activation of canonical PP2CAs requires destabilization or deactivation of ABA-independent RCARs. The WD40 protein RNA export factor 1 (RAE1) and DE-ETIOLATED 1 DAMAGED DNA-BINDING PROTEIN 1 ASSOCIATED1 (DDA1), which are proteins without functional sequence motifs, have been linked to promoting ubiquitin proteasome (UPS)-mediated degradation of group I RCARs via CULLIN4 (CUL4) E3 ligase complexes. RAE1 and DDA1 interact with RCAR1 and RCAR3, respectively, and promote their polyubiquitination *in planta* (Irigoyen et al., 2014; Li et al., 2018). DDA1 also interacts with RCAR10 in yeast but not with RCAR8 and RCAR9, suggesting that it may also control other ABA receptors, yet in a selective manner. Interaction of receptors with these proteins occur in the nucleus, but RAE1-RCAR1 complexes have also been observed in the cytosol. The plasma membrane (PM)-localized leucine-rich repeat receptor-like kinase (LRR-RLK) C-terminally Encoded Peptide Receptor 2 (CEPR2) was reported to interact with 9 out of 14 RCARs and has been shown to phosphorylate Ser54 of RCAR10, a strictly conserved residue among all ABA receptor N-*termini* (Yu et al., 2019). Nuclear Arabidopsis EL1-like (AEL) casein kinases interact and phosphorylate several RCARs. The highly conserved Ser136 and Ser182 phospho-residues of RCAR12 have been proposed to be under the control of AELs (Chen et al., 2018). Phosphorylation of RCAR by CEPR2 or AELs promotes their polyubiquitination, which in turn increases their degradation by the UPS (Chen et al., 2018; Yu et al., 2019).

In addition to 26S proteasome-mediated degradation, the ubiquitination of group III RCARs may result in receptor endocytosis mediated by clathrin proteins, such as RIPENING-REGULATED PROTEIN 1 (RRP1), and further trafficking to the Trans-Golgi Network (TGN) (Bueso et al., 2014; Hou and Shen, 2020). The proteins ALG-2 INTERACTING PROTEIN-X (ALIX), VACUOLAR PROTEIN SORTING23A (VPS23A), and FYVE-DOMAIN PROTEIN 1 (FYVE1) are part of the Endosomal Sorting Complexes Required for Transport (ESCRT) machinery and interact with ABA receptors at the TGN for sorting and delivery via multivesicular bodies to the vacuole for protein lysis (Belda-Palazon et al., 2016; Yu et al., 2016; García-León et al., 2019). As the different ESCRT components can interact with various but not always overlapping RCARs, this system possibly controls receptor abundance in a selective way. Plasma membrane-anchored C2-domain ABA-related (CAR) proteins have been suggested to possibly contribute to receptor sorting, as they recruit RCARs at the PM through physical interaction (Rodriguez et al., 2014). In agreement with a role in receptor removal, most of the corresponding mutants show increased ABA or stress sensitivity during germination, suggesting that these proteins are important in the control of germination, but some redundancy is often observed in multigenic families (**Table 1**). Yet, triple *car1 5 9* and *rrp1* show a slight reduction in ABA sensitivity during germination, possibly because CARs and RRP1 are also involved in controlling some other ABA core signalosome components. For example, RRP1 interacts with ABI1 possibly to mediate its endocytosis, and the phenotype of an *abi1* loss-of-function mutant is epistatic to *rrp1* (Hou and Shen, 2020). High levels of ABA activate ESCRT-mediated RCAR disposal but impede their phosphorylation by CEPR2 or AELs, possibly because ABA-bound receptors interact poorly with these kinases compared to apo-RCARs. ABA does not affect DDA1-RCAR interaction but, nevertheless, limits RCAR3 degradation, while it has no effect on the ability of RAE1 to control RCAR1 levels.

Besides marking RCARs for disposal, PTMs have been shown to directly regulate intrinsic RCAR activity. The kinase complex consisting of Target of Rapamycin (TOR) and the Regulatory-Associated Protein of TOR (RAPTOR) has been proposed to phosphorylate group III RCARs *in vivo* on a conserved residue located in the receptor’s ABA binding pocket (i.e. S119 of RCAR12) (Wang et al., 2018). The With No lysine (K) protein Kinase 8 (WNK8) was found to interact with RCAR11 in the cytosol and to phosphorylate it at several residues located close to the ABA binding site *in vitro* (Waadt et al., 2019). The Cytosolic ABA Receptor Kinase 1 (CARK1) interacts with all group III receptors in the cytosol and phosphorylates them *in vivo* on a conserved residue (i.e. T78 of RCAR11) that lies near the gate loop (Zhang et al., 2018; Li et al., 2019b). Both TOR and WNK8 act as negative regulators of ABA responses by reducing receptor affinity for ABA. In contrast, CARK1 promotes ABA signalling by stabilizing RCARs and promoting their association with PP2CAs. Germination phenotypes for the corresponding mutants align with the proposed function of these kinases (**Table 1**). While CARK1 promotes, TOR and WNK8 prevent overactivation of group III receptors. Hence, targeted phosphorylation at distinct residues counterbalances ABA sensitivity as a fine-tuning system.

### Looking after the central suppressors: Clade A PP2Cs

Cellular depletion of PP2CAs is an ABA- and RCAR-free alternative to promote ABA responses. Due to the high specificity of E3 ligases, as opposed to the broad RCAR inhibition at high ABA levels, targeted degradation also provides a way to precisely control the specific functions of each PP2CA (**Figure 5B**).

The PP2CA Interacting RING finger proteins 1 and 2 (PIR1 and PIR2) interact with AHG-type PP2CAs, including AHG1, but not ABI-type PP2CAs (Baek et al., 2019). The CULLIN RING LIGASES 3 (CRL3) substrate adaptors BTB/POZ AND MATH DOMAIN proteins 5 (BPM5), RING DOMAIN LIGASES 1 and 5 (RGLG1 and RGLG5), and CONSTITUTIVELY PHOTOMORPHOGENIC 1 (COP1) all interact with AHG3 and selectively with some ABI-type PP2CAs (i.e. RGLGs appear to not interact with ABI1 and HAB1) (Wu et al., 2016; Belda-Palazon et al., 2019; Julian et al., 2019; Chen et al., 2020). PUB13, a U-Box E3 ligase, and the complex involving UBIQUITIN-CONJUGATING ENZYME 27 (UBC27) and ABA INSENSITIVE RING PROTEIN 3 (AIRP3) ligase interact with ABI1 *in vivo*. These interactions promote ubiquitination of PP2CAs and their UPS-mediated degradation (Kong et al., 2015; Pan et al., 2020). Degradation of PP2CAs positively affects ABA signal transduction, and the relevance of such mechanisms for germination is exemplified by the reduced sensitivity of corresponding mutant seeds to ABA during germination (**Table 1**). High levels of ABA globally enhance PP2CA degradation mechanisms by E3 ligases. The formation of RCAR-PP2CA complexes facilitates target recognition by COP1, RGLGs, and PUB13, but in the case of the latter, ABA only seems to be required when ABI1 binds to group III RCARs, whereas simple interaction with monomeric receptors is sufficient (Kong et al., 2015; Wu et al., 2016; Chen et al., 2020). Moreover, RGLGs can be anchored to the PM through N-terminal glycine myristoylation, and ABA has been shown to inhibit the activity of N-Myristoyl Transferases (NMT), thus promoting RGLG translocation to the nucleus (Belda-Palazon et al., 2019). ABA-enhanced degradation of ABI1 by UBC27 possibly occurs because UBC27 is itself degraded by the UPS in non-stress conditions but stabilized in the presence of the hormone (Pan et al., 2020).

PP2CA activity is also regulated by reversible PTMs. External *stimuli* transduced by PM-localized proteins can be integrated directly into the core signalling through regulation of ABI-type PP2CAs in the cytosol. Their activity has been proposed to be controlled by several receptor-like kinases (RLKs). BRASSINOSTEROID INSENSITIVE 1-ASSOCIATED RECEPTOR KINASE 1 (BAK1) interacts with cytosolic PP2CAs (except for ABI2), and FERONIA (FER) interacts with ABI2 via the Guanine Exchange Factors (GEF)/ROP11 GTPase. Both systems have been proposed to enhance PP2CA activity (Yu et al., 2012; Deng et al., 2022). Two active TRANSMEMBRANE KINASES (TMKs), TMK1 and TMK4, and RECEPTOR DEAD KINASE 1 (RDK1), a putative leucine-rich repeat RLK with no kinase activity, interact with ABI-type PP2CAs (Kumar et al., 2017; Li et al., 2021). In addition to RLKs, the PM-localized Rapidly Accelerated Fibrosarcoma kinase 22 (RAF22) interacts with ABI1 (Sun et al., 2022). Phosphorylation of PP2CAs by BAK1, TMK1, or RAF22 has been proposed to temper their inhibition by RCARs, while phosphorylation by TMK4 or interaction with RDK1 negatively affect their function. Consequently, *bak1*, *tmk1*, and *raf22* mutants show an increased, while *tmk4* and *rdk1* show a reduced sensitivity to ABA during germination (**Table 1**) (Yu et al., 2012; Li et al., 2021; Yang et al., 2021; Deng et al., 2022; Sun et al., 2022). ABA promotes PP2CA interaction with RDK1 or TMKs at the PM and activates BAK1. RAF22 phosphorylation by active class III SnRK2s represses its function (Deng et al., 2022; Sun et al., 2022).

In the nucleus, the DEAD BOX RNA HELICASE 8 (RH8) interacts with AHG-type PP2CAs (including AHG1) and inhibits the phosphatase activity of recombinant AHG3. Mutants of *rh8* are hyposensitive to ABA during germination, and RH8 acts genetically as an upstream negative regulator of PP2CAs (Baek et al., 2018). Last, a particularity of the regulation of PP2CA function in seeds is the previously mentioned repression of AHG1 and AHG3 functions by DOG1.

### Gating the class III SnRK2 kinase hub

UPS disposal of the central ABA kinases can be mediated by the BTB (Broad complex, Tramtrack, and Bric-a-brac) E3 ligases, specifically BTB-A2.1 and BTB-A2.2, or HIGH OSMOTIC STRESS 15 (HOS15), a WD40-repeat protein, serving as substrate receptor for DDB1-CUL4 E3 ligase complexes (Ali et al., 2019; Cai et al., 2020). Interaction of SnRK2s with these proteins occurs in the nucleus and additionally in the cytosol for BTB-A2s (Ali et al., 2019; Cai et al., 2020). Triple *btb-a2.1 2 3* mutant seeds are more sensitive and overexpression lines of *BTB-A2s* are less sensitive to ABA during germination. However, seed ABA sensitivity of *hos15* mutant or overexpression lines is only slightly modified (**Table 1**). High ABA levels impede HOS15-SnRK2 interaction. In contrast, SnRK2 association with ABI1 and ABI2 promotes kinase recognition by HOS15. Thus, dephosphorylated SnRK2s appear as preferential substrates (Ali et al., 2019). At the PM, SnRK2.6 interacts with Senescence-Associated Subtilisin Protease (SASP) and is thus degraded (Wang et al., 2018). Although this mechanism has only been demonstrated for SnRK2.6, ABA-induced expression of *SASP*, increased ABA sensitivity of *sasp* mutant during germination, and high SASP protein abundance in dry seeds suggest that SAPS may also participate in the decay of other class III SnRK2s in germinating seeds (**Figure 5A**).

*In vitro*, the basal activity of class III SnRK2s leads to its auto-phosphorylation at several positions, including residues of the activation loop in the presence of ATP, but the auto-activation ability of SnRK2.2 and SnRK2.3 is lower compared to SnRK2.6 and, thus, requires trans-phosphorylation by other kinases (Belin et al., 2006; Boudsocq et al., 2007; Ng et al., 2011). SnRK2.6 can form heterodimers with other class III SnRK2s (Waadt et al., 2015). Possibly, SnRK2.6 trans-phosphorylates other isoforms, but it is not detected in dry seed tissues. Furthermore, ablation of PP2CA activity in *Physcomitrella patens* was found to increase the activity of PpSnRK2 only slightly, and its ABA-dependent activation was largely unaffected, indicating the existence of upstream kinases (Saruhashi et al., 2015; Shinozawa et al., 2019). Members of the plant B3 RAF kinase subfamily have been recently demonstrated to interact predominantly in the cytosol (and in the nucleus to a lesser extent) with class III SnRK2s and to phosphorylate conserved sites within the activation loop (S171, S175 and T176 on SnRK2.6) *in vivo* (Katsuta et al., 2020; Lin et al., 2020; Takahashi et al., 2020). Serine 171 of SnRK2.6 was not found to be auto-phosphorylated *in vitro* and is critical for ABA-dependent initial priming or re-activation after PP2CA-mediated inhibition. RAF3 and RAF4 (also called M3Kδ1 and M3Kδ7/ARK1) are present in seeds. Also, two Glycogen Synthase Kinases 3 (GSK3s): BRASSINOSTEROID INSENSITIVE 2 (BIN2) and its homolog BIN2-LIKE2 (BIL2) interact with SnRK2.2 and SnRK2.3 in the cytosol and nucleus and phosphorylate activation loop residues *in vitro* (Cai et al., 2014). Inhibitor-based repression of GSK3s leads to a reduction of SnRK2.3 T180 phosphorylation and activity *in vivo.* Mutant seeds for these kinases are less sensitive towards ABA during germination (**Table 1**). Besides its role in PP2CA activation, BAK1 represses SnRK2s via physical interaction and phosphorylation of a residue present in their catalytic domain (position T146 in SnRK2.6). As such, the germination phenotype of *snrk2.2 2.3* is epistatic to *bak1* (Deng et al., 2022). Type One Protein Phosphatase 1 (TOPP1), a Mn^2+^ phosphoprotein phosphatase, interacts with several SnRK2s, including all members of class III, in the nucleus (Shi, 2009; Hou et al., 2016). TOPP1 inhibits the activity of SnRK2.2 and SnRK2.3 by preventing their auto-phosphorylation *in vitro*, and the overall class III SnRK2 activity is enhanced in *topp1* seedlings exposed to ABA compared to the wild type (Hou et al., 2016). An atypical PP2C that does not cluster with any subclass of the family is the Kinase-Associated Protein Phosphatase (KAPP) (Schweighofer et al., 2004); all class III SnRK2s interact with KAPP at the plasma membrane. The *kapp* mutant seeds are hypersensitive to ABA during germination, hinting at a negative role of KAPP in ABA responses. A genetic analysis showed that KAPP acts upstream of SnRK2.2 and SnRK2.3, but due to the lack of biochemical analyses, it is unclear whether KAPP directly dephosphorylates SnRK2s (Lu et al., 2020).

### Corrupting the transcriptional effectors

KEEP ON GOING (KEG) is a RING-type E3 ligase that also contains a functional serine/threonine kinase domain. KEG localizes in the TGN, where it ubiquitinates ABI5 and mediates its degradation in the cytoplasm (Stone et al., 2006; Chen et al., 2013; Liu and Stone, 2013). In the nucleus, ABI5 forms a complex with the two CUL4-DDB1 substrate receptors DWD hypersensitive to ABA1 and ABA2 (DWA1 and DWA2), thus promoting ABI5 degradation (Lee et al., 2010). ABA impedes KEG function by promoting its degradation by the UPS; although ABA’s effect on DWAs is not obvious, the presumably ABA-activated form of ABI5 seems to be a preferential substrate (Liu and Stone, 2010; Gu and Innes, 2011; Liu and Stone, 2013). Double *dwa1 2* mutant seeds show a strong ABA-hypersensitive phenotype during germination, and post-germination growth of *keg* seedlings is arrested even without ABA (Stone et al., 2006; Lee et al., 2010). Hence, it was proposed that KEG maintains low levels of ABI5 in stress-free conditions, while DWA1 and DWA2 promote ABI5 degradation as an escape route after stress relief (Liu and Stone, 2013).

The small ubiquitin-like modifier (SUMO) E3 ligase SAP AND MIZ1 DOMAIN-CONTAINING LIGASE1 (SIZ1) modifies ABI5 at the residue K391. ABA sensitivity during germination is increased in *siz1* seeds. *SIZ1* functions genetically upstream of *ABI5*, and overexpressing lines of an ABI5 K391R neo-morphic variant are hypersensitive to ABA, indicating that SUMOylation of ABI5 by SIZ1 diminishes its function. Yet, the *siz1* mutant has lower levels of ABI5, indicating that SIZ1 leads to an inactivation but protects ABI5 from degradation (Miura et al., 2009).

The unknown function ABI FIVE BINDING PROTEINs (AFPs) were initially identified in a yeast two-hybrid screen using ABI5 as bait, but AFPs can also interact with some other group A bZIPs (Lopez-Molina et al., 2003; Garcia et al., 2008; Lynch et al., 2017). Single *afp1* or *afp2* mutants show increased ABA and stress sensitivity during germination (Lopez-Molina et al., 2003; Garcia et al., 2008; Chang et al., 2018). Homozygous *AFP2* overexpression seeds are green and desiccation intolerant, compromising their physiological characterization. Still, progenies from hemizygous overexpression lines display an ABA-blind phenotype during germination (Lynch et al., 2017; Lynch et al., 2022). Effects of non-functional *abi5* alleles are epistatic to *afp* during germination, and ABI5 protein is accumulated at higher levels in *afp* mutant seeds (Lopez-Molina et al., 2003; Garcia et al., 2008). Hence, it was proposed that AFPs negatively regulate ABA responses through ABI5 destabilization. However, AFP2 can still repress ABA signalling during germination in a genetic background stabilizing ABI5 protein, suggesting that degradation of this bZIP is not fully essential for AFP2 function (Lynch et al., 2022). AFP protein abundance is increased by stresses or ABA treatment but drops within hours after stress relief. As this dynamic pattern appears futile given the negative role of AFPs in ABA responses, it was proposed that AFPs accumulate in a latent form during stress and are activated and destabilized under favourable conditions to escape ABA-mediated growth inhibition (Lopez-Molina et al., 2003; Garcia et al., 2008; Lynch et al., 2017; Lynch et al., 2022).

The Calcium-Dependent Protein Kinases CPK4 and CPK11 phosphorylate ABF1 and ABF4 *in vitro* (Zhu et al., 2007). Similarly, Calcineurin B-like (CBL) Interacting Protein 26 (CIPK26) interacts with ABI5 and phosphorylates it *in vitro* (Lyzenga et al., 2013). BIN2 and BIL2 kinases, which promote ABA signalling by SnRK2 activation, can also directly interact with ABI5. BIN2 was shown to phosphorylate ABI5 at residues other than those phosphorylated by SnRK2s, and mutation of these residues affects ABA responses, suggesting that both types of kinases might be required for full activity of ABI5 (Hu and Yu, 2014). Germination phenotypes of mutant or overexpression lines indicate that all these kinases positively regulate ABA responses (**Table 1**). The Type 2A Phosphatase Associated Protein of 46 kD (TAP46) binds to ABI5 and maintains its phosphorylation levels possibly by preventing ABI5 inactivation by the PP2A catalytic subunit (Hu et al., 2014). Although direct dephosphorylation of ABI5 by this kind of phosphatase has not been demonstrated, this hypothesis aligns with the observation that ABI5 phosphorylation levels are increased after treatment with PP2A inhibitors. Moreover, *pp2a-c2* or regulatory subunit mutants show ABA-sensitivity phenotypes during germination (Kwak et al., 2002; Pernas et al., 2007; Hu et al., 2014; Née et al., 2017a). ABA sensitivity during germination of a *tap46* mutant or a *TAP46* overexpression line supports a positive role of this protein in ABA signalling (**Table 1**).

ABI5 was shown to shuttle between the cytosol and nucleus, but its activation by phosphorylation is not required for nuclear localization, as early studies showed it to localize in the nucleus even in the absence of stress (Lopez-Molina et al., 2002; Xia et al., 2019). Yet, the cytonuclear shuttling of ABI5-interacting proteins also regulates ABI5 function. For example, under optimal conditions, the WD40 protein exportin 1 (XPO1)-Interacting WD40 protein 1 (XIW1) is actively excluded from the nucleus by XPO1 transporters. Upon stress, XIW1 accumulates in the nucleus where it interacts with ABI5 to protect it from UPS degradation (Xu et al., 2019). Accordingly, *xiw1* seeds are less sensitive to ABA during germination. Besides its role in the ESCRT pathway, FYVE1 is phosphorylated upon stress by class III SnRK2s and translocates to the nucleus, where it interacts with the DNA-binding domain of ABI5 and ABF4, thereby inhibiting their transcriptional activities (Li et al., 2019a).

In brief, in addition to the inner ABA signalosome wiring, the signal can be modulated in a relatively specific manner at each layer of the pathway. The signal intensity can be increased or reduced through reversible PTMs and/or redirected into specific channels through targeted disposal of some core components. While reversible PTMs can rapidly affect the amplitude of the signal, targeted degradation is a more time- and energy-consuming but permanent way to modify ABA responses.

This complex molecular system can be roughly conceptualized as an electric circuit in which ABA is the source of a current that, if maintained at a sufficient intensity after traveling through series of core components, turns on germination arrest programs. The varying wirings between the main elements differ in terms of transduction efficiency (like electric resistance). Hence, in addition to a backup function, the different paths provide an adaptative chassis to control different levels of information. At each level of the system, the signal intensity can be continuously modulated by additional elements acting like potentiometers with different trimming levels (combinatorial PTMs) and other like fuses (disposal) that would require functional replacement to restart the system.

Although we focused only on the first regulation shell of the ABA core signalling, it is important to keep in mind that the regulators presented above are also subject to modification, including feedback regulation from core signalling and interaction between themselves. For example, class III SnRK2s control TOR activity (Wang et al., 2018); further, KEG interacts with CIPK26 to promote its degradation via UPS *in planta*, and, conversely, CIPK26 promotes the degradation of KEG by phosphorylation, suggesting a tight balancing between the activity of these regulators (Lyzenga et al., 2013; Lyzenga et al., 2017).

### Conclusions, limitations of the study, and future perspectives

As an essential molecular mediator of the plant growth-resilience trade-off, ABA is involved in many physiological aspects (Nakashima and Yamaguchi-Shinozaki, 2013; Yoshida et al., 2019). Within the green lineage, the gradually increasing complexity of the ABA signalosome up to large multigenic families in higher plants was key during land colonization (Sun et al., 2019; Sun et al., 2020). Indeed, the ability of higher plants to balance and specialize ABA responses using an optimized multiwire system based on an ancestral and minimal molecular backbone appears as a *sine qua non* to cope with stresses in a rapidly changing environment. Due to the central role of seeds for higher plant resilience, the functioning of ABA signalling in seeds may represent its maximum level of sophistication.

Large-scale data sets from recent proteomic, interaction, and functional studies fit squarely into the global frame bordered by prior genetic and biochemical reports. Despite that the information still represents only fragments, the integration this new information allows us to refine our understanding of ABA signalling in seeds to a higher precision level. The ABA signalling pathway in Arabidopsis seeds is characterized by (i) its specific composition including the presence of unorthodox components (**Figures 1**, **4**), (ii) the existence of singular information fluxes at the receptor and the PP2CA level (**Figures 2**, **3**), and (iii) the presence of a subset of side regulators directly affecting core component functions (**Figure 5**). Enlarging the picture of ABA signalling in seeds beyond the minimalist three-layered model allows one to further appreciate how seeds fine-tune ABA responses and, thus, germination progression. As a hallmark, the original composition of the ABA core signalosome in seeds suggests critical differences in its operation compared to other organs. For instance, in stomatal cells SnRK2.6 plays a critical role, while in seeds the other isoforms are preponderant. The cellular function of the most abundant PP2CA in seeds appears restricted to this organ, and, conversely, several known peripheral core signalosome regulators are absent in seeds.

The incorporation of several research lines into a coherent picture of ABA signalling in seeds is a challenging task that is further complicated by some technical differences in the experimentation procedure between independent laboratories. To provide constructive feedback from our analysis, we recommend adding well-characterized lines for the described phenotype (i.e. *abi5* mutant for reduced ABA sensitivity) as internal controls in addition to wild type genotypes when performing germination assays. Also, we recommend using a broad scale of ABA doses rather than a single concentration to gain a better resolution. Indeed, reference genotypes are extremely valuable to gauge relative effects and to integrate information into a coherent model. We also note that the term “ABA insensitive” is widely used to illustrate a reduced sensitivity ranging from a half to hundreds of micromolar of ABA. Similarly, dormancy is often inappropriately used to describe germination arrest under stress conditions, and acquisition of autotrophy (cotyledon greening) is confused with germination *per se*. Although all these traits are controlled by ABA, they are clearly distinct developmental processes underlying different molecular mechanisms. To avoid confusion, we advocate for the use of terminology that adequately reflects the actual experimentation setup.

As a limitation of this study, it should be kept in mind that the basis of this meta-analysis is the dry seed proteome of the *Arabidopsis thaliana* ecotype Col-0 obtained under common laboratory cultivation parameters. Conditions experienced during seed maturation deeply affect seed germination quality. Consequently, it can be expected that different maturation conditions will affect the initial composition of the seed ABA core signalosome. Because germination behaviour is a strong determinant of plant adaptation, the same can also be assumed within Arabidopsis ecotypes due to natural variation or for other seed species. Seed proteomic studies as well as characterization of ABA signalosome components in other species will, on the one hand, help to precisely modify seed quality in crops and, on the other hand, will allow one to directly address the basis for adaptation of germination behaviours during speciation at molecular levels. Also, as many functional assays (i.e. interaction screens) were performed outside of seed tissues, these data possibly abstract relevant differences in terms of cellular context. Paired interactions of side regulators with core components were not always addressed family wide. This means that the nevertheless already apparent concept of isoform functional specialization may strongly benefit from further refining.

Though starting from the static situation of the dry seed proteome, our analysis indicates a dynamic remodelling of information fluxes during imbibition. Understanding how different inner and environmental inputs are specifically transcribed into distinct molecular events that modify the ABA signalosome operation to control the progression of germination is an open question in seed biology. We foresee that studies combining *in semilla* proteomic, molecular, and genetic approaches will greatly help to fill this gap and will possibly lead to the discovery of new mechanisms in the field of germination and ABA signalling.

## Data availability statement

The original contributions presented in the study are included in the article/**Supplementary Material**. Further inquiries can be directed to the corresponding author.

## Author contributions

GN conceived the study with inputs from TK. GN and TK collected information, performed analysis, prepared figures, and drafted the manuscript. GN wrote the final version. Both authors approved the submitted version.
