# Supplementary material for: Dry side of the core: a meta-analysis addressing the original nature of the ABA signalosome at the onset of seed imbibition
Source: Front Plant Sci. 2023 Jul 5;14:1192652. doi: 10.3389/fpls.2023.1192652 (PMC10354442; doi:10.3389/fpls.2023.1192652)
Supplement: Supplementary file 1 [file Table_1.docx]

**Supplemental Table 1**: **Identifiers of proteins belonging to multi-genic families involved in ABA signalling in Arabidopsis**. UniProtKB identifiers of protein sequences, which were used to generate phylogenetic tress of Figure 1B based on ClustalOmega alignment.

| **Protein** | **UniProt identifier** |
| --- | --- |
| RCAR1 | Q84MC7 |
| RCAR2 | Q1ECF1 |
| RCAR3 | Q9FGM1 |
| RCAR4 | Q8H1R0 |
| RCAR5 | Q9FJ50 |
| RCR6 | Q9FJ49 |
| RCAR7 | Q9SN51 |
| RCAR8 | Q9FLB1 |
| RCAR9 | Q8S8E3 |
| RCAR10 | O80920 |
| RCAR11 | O49686 |
| RCAR12 | Q8VZS8 |
| RCAR13 | Q9SSM7 |
| RCAR14 | O80992 |
| ABI1 | P49597 |
| ABI2 | O04719 |
| HAB1 | Q9LNP9 |
| HAB2 | Q9CAJ0 |
| HAI1 | Q9FIF5 |
| HAI2 | Q9LNW3 |
| HAI3 | Q9ZW21 |
| AHG1 | Q9FLI3 |
| AHG3 | P49598 |
| SnRK2.1 | P43292 |
| SnRK2.2 | Q39192 |
| SnRK2.3 | Q39193 |
| SnRK2.4 | P43291 |
| SnRK2.5 | Q9FFP9 |
| SnRK2.6 | Q940H6 |
| SnRK2.7 | Q9SMQ4 |
| SnRK2.8 | Q9M9E9 |
| SnRK2.9 | O64812 |
| SnRK2.10 | Q9C958 |
| bZIP15 | Q9FMM7 |
| ABF1 | Q9M7Q5 |
| ABF2 | Q9M7Q4 |
| ABF3 | Q9M7Q3 |
| ABF4 | Q9M7Q2 |
| EEL | Q9C5Q2 |
| AREB3 | Q9LES3 |
| DPBF2 | Q8RYD6 |
| ABI5 | Q9SJN0 |
| bZIP13 | Q9FNB9 |
| GBF4 | P42777 |
| FD | Q84JK2 |
| FDP | Q7PCC6 |

**Supplemental Table 2: Proteins described to act as peripheral regulators of the core ABA signalling pathway in *A. thaliana*.** Proteins are categorized according to protein classes or larger complexes they are involved in. Their respective targets of the core ABA components are listed. More precise information about target redundancy and specificity is found in the main text. According to dry seed proteome data, retrieved from Mergner et al. 2020, proteins are categorized as present or absent in this tissue.

| **Protein** | **Protein class** | **Target** | **Present in dry seed** | **Source(s)** |
| --- | --- | --- | --- | --- |
| CARK1 | Kinase | RCARs | Yes | (X. Li et al., 2019; Zhang et al., 2018) |
| BAK1 | Kinase | PP2Cs | Yes | (Deng et al., 2022) |
| RPK1 | Kinase | SnRK2s | No | (Shang et al., 2020) |
| TMK1 | Kinase | PP2Cs | Yes | (L. Li et al., 2021; Yang et al., 2021) |
| TMK4 | Kinase | PP2Cs | Yes | (L. Li et al., 2021) |
| M3Kδ1/RAF3 | Kinase | SnRK2s | Yes | (Takahashi et al., 2020) |
| ARK1/M3Kδ7/ RAF4 | Kinase | SnRK2s | Yes | (Katsuta et al., 2020; Takahashi et al., 2020) |
| ARK2/M3Kδ6/RAF5 | Kinase | SnRK2s | No | (Katsuta et al., 2020; Takahashi et al., 2020) |
| ARK3/RAF6 | Kinase | SnRK2s | No | (Katsuta et al., 2020) |
| RAF10 | Kinase | SnRK2s, bZIPs | No | (S. J. Lee et al., 2015; Nguyen et al., 2019) |
| RAF22 | Kinase | PP2Cs | Yes | (Sun et al., 2022) |
| CPK4 | Kinase | bZIPs | Yes | (Zhu et al., 2007) |
| CPK11 | Kinase | bZIPs | Yes | (Zhu et al., 2007) |
| CEPR2 | Kinase | RCARs | Yes | (Z. Yu et al., 2019) |
| AEL1 | Kinase | RCARs | Yes | (H. H. Chen et al., 2018) |
| AEL2 | Kinase | RCARs | No | (Chen et al., 2018) |
| AEL3 | Kinase | RCARs | Yes | (Chen et al., 2018) |
| AEL4 | Kinase | RCARs | Yes | (Chen et al., 2018) |
| RDK1 | Kinase | PP2Cs | Yes | (Kumar et al., 2017) |
| WNK8 | Kinase | RCARs | Yes | (Waadt et al., 2019) |
| FER | Kinase | PP2Cs | Yes | (J. Chen et al., 2016; F. Yu et al., 2012) |
| BIN2 | Kinase | SnRK2s, bZIPs | Yes | (Z. Cai et al., 2014) |
| BIL2 | Kinase | SnRK2s, bZIPs | Yes | (Z. Cai et al., 2014) |
| CIPK26 | Kinase | bZIPs | Yes | (Lyzenga et al., 2013) |
| CIPK11/PKS5 | Kinase | bZIPs | No | (Zhou et al., 2015) |
| TOR | Kinase | bZIPs | Yes | (P. Wang et al., 2018) |
| TOPP1 | Phosphatase | SnRK2s | Yes | (Y. J. Hou et al., 2016) |
| KAPP | Phosphatase | SnRK2s | Yes | (Lu et al., 2020) |
| PIR1 | E3 Ligase | PP2Cs | Yes | (Baek et al., 2019) |
| PIR2 | E3 Ligase | PP2Cs | Yes | (Baek et al., 2019) |
| DDA1 | E3 Ligase | RCARs | Yes | (Irigoyen et al., 2014) |
| RSL1 | E3 Ligase | RCARs | No | (Bueso et al., 2014) |
| KEG | E3 Ligase | bZIPs | Yes | (Stone et al., 2006; Gu and Innes, 2011; Liu and Stone, 2013) |
| COP1 | E3 Ligase | PP2Cs | Yes | (Q. Chen et al., 2020) |
| PUB22 | E3 Ligase | RCARs | No | (Zhao et al., 2017) |
| PUB23 | E3 Ligase | RCARs | No | (Zhao et al., 2017) |
| PUB12 | E3 Ligase | PP2Cs | No | (Zhao et al., 2017) |
| PUB13 | E3 Ligase | PP2Cs | Yes | (Zhao et al., 2017) |
| RFA4 | E3 Ligase | RCARs | No | (Fernandez et al., 2020) |
| RFA1 | E3 Ligase | RCARs | No | (Fernandez et al., 2020) |
| AIRP3 | E3 Ligase | PP2Cs | Yes | (Pan et al., 2020) |
| RGLG1 | E3 Ligase | PP2Cs | Yes | (Belda-Palazon et al., 2019; Wu et al., 2016) |
| RGLG5 | E3 Ligase | PP2Cs | Yes | (Wu et al., 2016) |
| RIFP1 | E3 Ligase F Box | RCARs | No | (Y. Li et al., 2016) |
| RAE1 | DWD protein | RCARs | Yes | (D. Li et al., 2018) |
| DWA1 | DWD proteins | bZIPs | Yes | (J. H. Lee et al., 2010) |
| DWA2 | DWD proteins | bZIPs | Yes | (J. H. Lee et al., 2010) |
| PP2B11 | E3 ligase complex | SnRK2s | No | (Cheng et al., 2017) |
| BPM3 | E3 ligase complex | PP2Cs | No | (Julian et al., 2019) |
| BPM5 | E3 ligase complex | PP2Cs | Yes | (Julian et al., 2019) |
| BTB-A2.1 | E3 ligase complex | SnRK2s | Yes | (G. Cai et al., 2020) |
| BTB-A2.2 | E3 ligase complex | SnRK2s | Yes | (G. Cai et al., 2020) |
| BTB-A2.3 | E3 ligase complex | SnRK2s | No | (G. Cai et al., 2020) |
| SIZ1 | SUMOylase | bZIPs | Yes | (Miura et al., 2009) |
| ALIX | ESCRT component | RCARs | Yes | (García-León et al., 2019) |
| FYVE1/FREE1 | ESCRT component | RCARs | Yes | (Belda-Palazon et al., 2016; H. Li et al., 2019) |
| VPS23A | ESCRT component | RCARs | Yes | (F. Yu et al., 2016) |
| CAR1 | Calcium-dependent lipid-binding protein | RCARs | Yes | (Rodriguez et al., 2014) |
| CAR4 | Calcium-dependent lipid-binding protein | RCARs | No | (Rodriguez et al., 2014) |
| CAR5 | Calcium-dependent lipid-binding protein | RCARs | Yes | (Rodriguez et al., 2014) |
| CAR9 | Calcium-dependent lipid-binding protein | RCARs | Yes | (Rodriguez et al., 2014) |
| UBC26 | Ubiquitin-conjugating enzyme | RCARs | No | (Fernandez et al., 2020) |
| UBC27 | Ubiquitin-conjugating enzyme | PP2Cs | Yes | (Pan et al., 2020) |
| XIW1 | WD40 | bZIPs | Yes | (Xu et al., 2019) |
| ABT | WD40 | RCARs | No | (Z. Wang et al., 2020) |
| HOS15 | WD40 | SnRK2s | Yes | (Ali et al., 2019) |
| ABD1 | WD40 | bZIPs | No | (Seo et al., 2014) |
| AFP1 | NINJA like protein | bZIPs | Yes | (Garcia et al., 2008; Lopez-Molina et al., 2003) |
| AFP2 | NINJA like protein | bZIPs | Yes | (Garcia et al., 2008) |
| AFP3 | NINJA like protein | bZIPs | No | (Garcia et al., 2008) |
| AFP4 | NINJA like protein | bZIPs | No | (Garcia et al., 2008) |
| SAB1 | RCC1 protein | bZIPs | No | (Ji et al., 2019) |
| SASP | Protease | SnRKs | Yes | (Q. Wang et al., 2018) |
| TAP46 | Unknown | bZIPs | Yes | (Hu et al., 2014) |
| DOG1 | DOG1-family protein | PP2Cs | Yes | (Née et al., 2017; Nishimura et al., 2018) |
| EAR1 | Fantastic four protein | PP2Cs | No | (K. Wang et al., 2018) |
| ROP11 | GTPase | PP2Cs | Yes | (J. Chen et al., 2016; F. Yu et al., 2012) |
| AtI2 | phosphatase inhibitor protein | SnRK2 | No | (Y. J. Hou et al., 2016) |
| SCD2/RRP1 | Coiled-coil protein | RCARs | Yes | (B. Hou & Shen, 2020) |
| RH8 | DEAD Box RNA Helicase | PP2Cs | Yes | (Baek et al., 2018) |

**References:**

Ali, A., Kim, J. K., Jan, M., Khan, H. A., Khan, I. U., Shen, M., Park, J., Lim, C. J., Hussain, S., Baek, D., Wang, K., Chung, W. S., Rubio, V., Lee, S. Y., Gong, Z., Kim, W. Y., Bressan, R. A., Pardo, J. M., & Yun, D. J. (2019). Rheostatic Control of ABA Signaling through HOS15-Mediated OST1 Degradation. *Molecular Plant*, *12*(11), 1447–1462. https://doi.org/10.1016/j.molp.2019.08.005

Baek, W., Lim, C. W., & Lee, S. C. (2018). A DEAD-box RNA helicase, RH8, is critical for regulation of ABA signalling and the drought stress response via inhibition of PP2CA activity. *Plant Cell and Environment*, *41*(7), 1593–1604. https://doi.org/10.1111/pce.13200

Baek, W., Lim, C. W., Luan, S., & Lee, S. C. (2019). The RING finger E3 ligases PIR1 and PIR2 mediate PP2CA degradation to enhance abscisic acid response in Arabidopsis. *Plant Journal*, *100*(3), 473–486. https://doi.org/10.1111/tpj.14507

Belda-Palazon, B., Julian, J., Coego, A., Wu, Q., Zhang, X., Batistic, O., Alquraishi, S. A., Kudla, J., An, C., & Rodriguez, P. L. (2019). ABA inhibits myristoylation and induces shuttling of the RGLG1 E3 ligase to promote nuclear degradation of PP2CA. *Plant Journal*, *98*(5), 813–825. https://doi.org/10.1111/tpj.14274

Belda-Palazon, B., Rodriguez, L., Fernandez, M. A., Castillo, M. C., Anderson, E. M., Gao, C., Gonzalez-Guzman, M., Peirats-Llobet, M., Zhao, Q., de Winne, N., Gevaert, K., de Jaeger, G., Jiang, L., Leï¿½n, J., Mullen, R. T., & Rodriguez, P. L. (2016). FYVE1/FREE1 interacts with the PYL4 ABA receptor and mediates its delivery to the vacuolar degradation pathway. *Plant Cell*, *28*(9), 2291–2311. https://doi.org/10.1105/tpc.16.00178

Bueso, E., Rodriguez, L., Lorenzo-Orts, L., Gonzalez-Guzman, M., Sayas, E., Muñoz-Bertomeu, J., Ibañez, C., Serrano, R., & Rodriguez, P. L. (2014). The single-subunit RING-type E3 ubiquitin ligase RSL1 targets PYL4 and PYR1 ABA receptors in plasma membrane to modulate abscisic acid signaling. *Plant Journal*, *80*(6), 1057–1071. https://doi.org/10.1111/tpj.12708

Cai, G., Wang, Y., Tu, G., Chen, P., Luan, S., & Lan, W. (2020). Type A2 BTB Members Decrease the ABA Response during Seed Germination by Affecting the Stability of SnRK2.3 in Arabidopsis. *International Journal of Molecular Sciences*, *21*(9). https://doi.org/10.3390/ijms21093153

Cai, Z., Liu, J., Wang, H., Yang, C., Chen, Y., Li, Y., Pan, S., Dong, R., Tang, G., de Dios Barajas-Lopez, J., Fujii, H., & Wang, X. (2014). GSK3-like kinases positively modulate abscisic acid signaling through phosphorylating subgroup III SnRK2s in Arabidopsis. *Proceedings of the National Academy of Sciences of the United States of America*, *111*(26), 9651–9656. https://doi.org/10.1073/pnas.1316717111

Chen, H. H., Qu, L., Xu, Z. H., Zhu, J. K., & Xue, H. W. (2018). EL1-like Casein Kinases Suppress ABA Signaling and Responses by Phosphorylating and Destabilizing the ABA Receptors PYR/PYLs in Arabidopsis. *Molecular Plant*, *11*(5), 706–719. https://doi.org/10.1016/j.molp.2018.02.012

Chen, J., Yu, F., Liu, Y., Du, C., Li, X., Zhu, S., Wang, X., Lan, W., Rodriguez, P. L., Liu, X., Li, D., Chen, L., & Luan, S. (2016). FERONIA interacts with ABI2-type phosphatases to facilitate signaling cross-talk between abscisic acid and RALF peptide in Arabidopsis. *Proceedings of the National Academy of Sciences of the United States of America*, *113*(37), E5519–E5527. https://doi.org/10.1073/pnas.1608449113

Chen, Q., Bai, L., Wang, W., Shi, H., Botella, J. R., Zhan, Q., Liu, K., Yang, H., & Song, C. (2020). COP1 Promotes ABA‐Induced Stomatal Closure by Modulating the Abundance of ABI/HAB and AHG3 Phosphatases. In *New Phytologist*. https://doi.org/10.1111/nph.17001

Cheng, C., Wang, Z., Ren, Z., Zhi, L., Yao, B., Su, C., Liu, L., & Li, X. (2017). SCFAtPP2-B11modulates ABA signaling by facilitating SnRK2.3 degradation in Arabidopsis thaliana. *PLoS Genetics*, *13*(8). https://doi.org/10.1371/journal.pgen.1006947

Deng, J., Kong, L., Zhu, Y., Pei, D., Chen, X., Wang, Y., Qi, J., Song, C., Yang, S., & Gong, Z. (2022). BAK1 plays contrasting roles in regulating abscisic acid-induced stomatal closure and abscisic acid-inhibited primary root growth in Arabidopsis. *Journal of Integrative Plant Biology*, *64*(6), 1264–1280. https://doi.org/https://doi.org/10.1111/jipb.13257

Fernandez, M. A., Belda-Palazon, B., Julian, J., Coego, A., Lozano-Juste, J., Iñigo, S., Rodriguez, L., Bueso, E., Goossens, A., & Rodriguez, P. L. (2020). RBR-Type E3 Ligases and the Ubiquitin-Conjugating Enzyme UBC26 Regulate Abscisic Acid Receptor Levels and Signaling1  [OPEN]. *Plant Physiology*, *182*(4), 1723–1742. https://doi.org/10.1104/pp.19.00898

Garcia, M. E., Lynch, T., Peeters, J., Snowden, C., & Finkelstein, R. (2008). A small plant-specific protein family of ABI five binding proteins (AFPs) regulates stress response in germinating Arabidopsis seeds and seedlings. *Plant Molecular Biology*, *67*(6), 643–658. https://doi.org/10.1007/s11103-008-9344-2

García-León, M., Cuyas, L., Abd El-Moneim, D., Rodriguez, L., Belda-Palazón, B., Sanchez-Quant, E., Fernández, O., Roux, B., María Zamarreño, Á., María García-Mina, J., Nussaume, L., Rodriguez, P. L., Paz-Ares, J., Leonhardt, N., & Rubio, V. (2019). Arabidopsis ALIX regulates stomatal aperture and turnover of abscisic acid receptors. *Plant Cell*, *31*(10), 2411–2429. https://doi.org/10.1105/tpc.19.00399

Hou, B., & Shen, Y. (2020). A Clathrin-Related Protein, SCD2/RRP1, Participates in Abscisic Acid Signaling in Arabidopsis. *Frontiers in Plant Science*, *11*(June), 1–14. https://doi.org/10.3389/fpls.2020.00892

Hou, Y. J., Zhu, Y., Wang, P., Zhao, Y., Xie, S., Batelli, G., Wang, B., Duan, C. G., Wang, X., Xing, L., Lei, M., Yan, J., Zhu, X., & Zhu, J. K. (2016). Type One Protein Phosphatase 1 and Its Regulatory Protein Inhibitor 2 Negatively Regulate ABA Signaling. *PLoS Genetics*, *12*(3), 1–22. https://doi.org/10.1371/journal.pgen.1005835

Hu, R., Zhu, Y., Shen, G., & Zhang, H. (2014). TAP46 plays a positive role in the ABSCISIC ACID INSENSITIVE5-regulated gene expression in Arabidopsis. *Plant Physiology*, *164*(2), 721–734. https://doi.org/10.1104/pp.113.233684

Irigoyen, M. L., Iniesto, E., Rodriguez, L., Puga, M. I., Yanagawa, Y., Pick, E., Strickland, E., Paz-Ares, J., Wei, N., De Jaeger, G., Rodriguez, P. L., Deng, X. W., & Rubio, V. (2014). Targeted degradation of abscisic acid receptors is mediated by the ubiquitin ligase substrate adaptor DDA1 in Arabidopsis. *The Plant Cell*, *26*(2), 712–728. https://doi.org/10.1105/tpc.113.122234

Ji, H., Wang, S., Cheng, C., Li, R., Wang, Z., Jenkins, G. I., Kong, F., & Li, X. (2019). The RCC1 family protein SAB1 negatively regulates ABI5 through multidimensional mechanisms during postgermination in Arabidopsis. *New Phytologist*, *222*(2), 907–922. https://doi.org/10.1111/nph.15653

Julian, J., Coego, A., Lozano-Juste, J., Lechner, E., Wu, Q., Zhang, X., Merilo, E., Belda-Palazon, B., Park, S. Y., Cutler, S. R., An, C., Genschik, P., & Rodriguez, P. L. (2019). The MATH-BTB BPM3 and BPM5 subunits of Cullin3-RING E3 ubiquitin ligases target PP2CA and other clade A PP2Cs for degradation. *Proceedings of the National Academy of Sciences of the United States of America*, *116*(31), 15725–15734. https://doi.org/10.1073/pnas.1908677116

Katsuta, S., Masuda, G., Bak, H., Shinozawa, A., Kamiyama, Y., Umezawa, T., Takezawa, D., Yotsui, I., Taji, T., & Sakata, Y. (2020). Arabidopsis Raf-like kinases act as positive regulators of subclass III SnRK2 in osmostress signaling. *Plant Journal*, *103*(2), 634–644. https://doi.org/10.1111/tpj.14756

Kumar, D., Kumar, R., Baek, D., Hyun, T. K., Chung, W. S., Yun, D. J., & Kim, J. Y. (2017). Arabidopsis thaliana RECEPTOR DEAD KINASE1 Functions as a Positive Regulator in Plant Responses to ABA. *Molecular Plant*, *10*(2), 223–243. https://doi.org/10.1016/j.molp.2016.11.011

Lee, J. H., Yoon, H. J., Terzaghi, W., Martinez, C., Dai, M., Li, J., Byun, M. O., & Deng, X. W. (2010). DWA1 and DWA2, two Arabidopsis DWD protein components of CUL4-based E3 ligases, act together as negative regulators in ABA signal transduction. *Plant Cell*, *22*(6), 1716–1732. https://doi.org/10.1105/tpc.109.073783

Lee, S. J., Lee, M. H., Kim, J. Il, & Kim, S. Y. (2015). Arabidopsis putative MAP kinase kinase kinases raf10 and raf11 are positive regulators of seed dormancy and ABA response. *Plant and Cell Physiology*, *56*(1), 84–97. https://doi.org/10.1093/pcp/pcu148

Li, D., Zhang, L., Li, X., Kong, X., Wang, X., Li, Y., Liu, Z., Wang, J., Li, X., & Yang, Y. (2018). AtRAE1 is involved in degradation of ABA receptor RCAR1 and negatively regulates ABA signalling in Arabidopsis. *Plant Cell and Environment*, *41*(1), 231–244. https://doi.org/10.1111/pce.13086

Li, H., Li, Y., Zhao, Q., Li, T., Wei, J., Li, B., Shen, W., Yang, C., Zeng, Y., Rodriguez, P. L., Zhao, Y., Jiang, L., Wang, X., & Gao, C. (2019). The plant ESCRT component FREE1 shuttles to the nucleus to attenuate abscisic acid signalling. *Nature Plants*, *5*(5), 512–524. https://doi.org/10.1038/s41477-019-0400-5

Li, L., Li, B., Zhu, S., Wang, L., Song, L., Chen, J., Ming, Z., Liu, X., Li, X., & Yu, F. (2021). TMK4 receptor kinase negatively modulates ABA signaling by phosphorylating ABI2 and enhancing its activity. *Journal of Integrative Plant Biology*, *63*(6), 1161–1178. https://doi.org/https://doi.org/10.1111/jipb.13096

Li, X., Kong, X., Huang, Q., Zhang, Q., Ge, H., Zhang, L., Li, G., Peng, L., Liu, Z., Wang, J., Li, X., & Yang, Y. (2019). CARK1 phosphorylates subfamily III members of ABA receptors. *Journal of Experimental Botany*, *70*(2), 519–528. https://doi.org/10.1093/jxb/ery374

Li, Y., Zhang, L., Li, D., Liu, Z., Wang, J., Li, X., & Yang, Y. (2016). The Arabidopsis F-box E3 ligase RIFP1 plays a negative role in abscisic acid signalling by facilitating ABA receptor RCAR3 degradation. *Plant, Cell & Environment*, *39*(3), 571–582. https://doi.org/https://doi.org/10.1111/pce.12639

Lopez-Molina, L., Mongrand, S., Kinoshita, N., & Chua, N.-H. (2003). AFP is a novel negative regulator of ABA signaling that promotes ABI5 protein degradation. *Genes & Development*, *17*(3), 410–418. https://doi.org/10.1101/gad.1055803

Lu, K., Zhang, Y. D., Zhao, C. F., Zhou, L. H., Zhao, Q. Y., Chen, T., & Wang, C. L. (2020). The Arabidopsis kinase-associated protein phosphatase KAPP, interacting with protein kinases SnRK2.2/2.3/2.6, negatively regulates abscisic acid signaling. *Plant Molecular Biology*, *102*(1–2), 199–212. https://doi.org/10.1007/s11103-019-00941-8

Lyzenga, W. J., Liu, H., Schofield, A., Muise-Hennessey, A., & Stone, S. L. (2013). Arabidopsis CIPK26 interacts with KEG, components of the ABA signalling network and is degraded by the ubiquitin-proteasome system. *Journal of Experimental Botany*, *64*(10), 2779–2791. https://doi.org/10.1093/jxb/ert123

Miura, K., Lee, J., Jin, J. B., Yoo, C. Y., Miura, T., & Hasegawa, P. M. (2009). Sumoylation of ABI5 by the Arabidopsis SUMO E3 ligase SIZ1 negatively regulates abscisic acid signaling. *Proceedings of the National Academy of Sciences of the United States of America*, *106*(13), 5418–5423. https://doi.org/10.1073/pnas.0811088106

Née, G., Kramer, K., Nakabayashi, K., Yuan, B., Xiang, Y., Miatton, E., Finkemeier, I., & Soppe, W. J. J. (2017). DELAY of GERMINATION1 requires PP2C phosphatases of the ABA signalling pathway to control seed dormancy /631/449/2679/2683 /631/449/2653 article. *Nature Communications*, *8*(1), 1–8. https://doi.org/10.1038/s41467-017-00113-6

Nguyen, Q. T. C., Lee, S. J., Choi, S. W., Na, Y. J., Song, M. R., Hoang, Q. T. N., Sim, S. Y., Kim, M. S., Kim, J. Il, Soh, M. S., & Kim, S. Y. (2019). Arabidopsis Raf-Like Kinase Raf10 Is a Regulatory Component of Core ABA Signaling. *Molecules and Cells*, *42*(9), 646–660. https://doi.org/10.14348/molcells.2019.0173

Nishimura, N., Tsuchiya, W., Moresco, J. J., Hayashi, Y., Satoh, K., Kaiwa, N., Irisa, T., Kinoshita, T., Schroeder, J. I., Yates, J. R., Hirayama, T., & Yamazaki, T. (2018). Control of seed dormancy and germination by DOG1-AHG1 PP2C phosphatase complex via binding to heme. *Nature Communications*, *9*(1), 2132. https://doi.org/10.1038/s41467-018-04437-9

Pan, W., Lin, B., Yang, X., Liu, L., Xia, R., Li, J., Wu, Y., & Xie, Q. (2020). The UBC27-AIRP3 ubiquitination complex modulates ABA signaling by promoting the degradation of ABI1 in Arabidopsis. *Proceedings of the National Academy of Sciences of the United States of America*. https://doi.org/10.1073/pnas.2007366117

Rodriguez, L., Gonzalez-Guzman, M., Diaz, M., Rodrigues, A., Izquierdo-Garcia, A. C., Peirats-Llobet, M., Fernandez, M. A., Antoni, R., Fernandez, D., Marquez, J. A., Mulet, J. M., Albert, A., & Rodriguez, P. L. (2014). C2-Domain Abscisic Acid-Related Proteins Mediate the Interaction of PYR/PYL/RCAR Abscisic Acid Receptors with the Plasma Membrane and Regulate Abscisic Acid Sensitivity in Arabidopsis    . *The Plant Cell*, *26*(12), 4802–4820. https://doi.org/10.1105/tpc.114.129973

Seo, K. I., Lee, J. H., Nezames, C. D., Zhong, S., Song, E., Byun, M. O., & Deng, X. W. (2014). ABD1 is an Arabidopsis DCAF substrate receptor for CUL4-DDB1-based E3 ligases that acts as a negative regulator of abscisic acid signaling. *Plant Cell*, *26*(2), 695–711. https://doi.org/10.1105/tpc.113.119974

Shang, Y., Yang, D., Ha, Y., Shin, H. Y., & Nam, K. H. (2020). Receptor-like protein kinases RPK1 and BAK1 sequentially form complexes with the cytoplasmic kinase OST1 to regulate ABA-induced stomatal closure. *Journal of Experimental Botany*, *71*(4), 1491–1502. https://doi.org/10.1093/jxb/erz489

Sun, Z., Feng, Z., Ding, Y., Qi, Y., Jiang, S., Li, Z., Wang, Y., Qi, J., Song, C., Yang, S., & Gong, Z. (2022). RAF22, ABI1 and OST1 form a dynamic interactive network that optimizes plant growth and responses to drought stress in <em>Arabidopsis</em>. *Molecular Plant*, *15*(7), 1192–1210. https://doi.org/10.1016/j.molp.2022.06.001

Takahashi, Y., Zhang, J., Hsu, P. K., Ceciliato, P. H. O., Zhang, L., Dubeaux, G., Munemasa, S., Ge, C., Zhao, Y., Hauser, F., & Schroeder, J. I. (2020). MAP3Kinase-dependent SnRK2-kinase activation is required for abscisic acid signal transduction and rapid osmotic stress response. *Nature Communications*, *11*(1). https://doi.org/10.1038/s41467-019-13875-y

Waadt, R., Jawurek, E., Hashimoto, K., Li, Y., Scholz, M., Krebs, M., Czap, G., Hong-Hermesdorf, A., Hippler, M., Grill, E., Kudla, J., & Schumacher, K. (2019). Modulation of ABA responses by the protein kinase WNK8. *FEBS Letters*, *593*(3), 339–351. https://doi.org/https://doi.org/10.1002/1873-3468.13315

Wang, K., He, J., Zhao, Y., Wu, T., Zhou, X., Ding, Y., Kong, L., Wang, X., Wang, Y., Li, J., Song, C. P., Wang, B., Yang, S., Zhu, J. K., & Gong, Z. (2018). EAR1 negatively regulates ABA signaling by enhancing 2C protein phosphatase activity. *Plant Cell*, *30*(4), 815–834. https://doi.org/10.1105/tpc.17.00875

Wang, P., Zhao, Y., Li, Z., Hsu, C. C., Liu, X., Fu, L., Hou, Y. J., Du, Y., Xie, S., Zhang, C., Gao, J., Cao, M., Huang, X., Zhu, Y., Tang, K., Wang, X., Tao, W. A., Xiong, Y., & Zhu, J. K. (2018). Reciprocal Regulation of the TOR Kinase and ABA Receptor Balances Plant Growth and Stress Response. *Molecular Cell*, *69*(1), 100-112.e6. https://doi.org/10.1016/j.molcel.2017.12.002

Wang, Q., Guo, Q., Guo, Y., Yang, J., Wang, M., Duan, X., Niu, J., Liu, S., Zhang, J., Lu, Y., Hou, Z., Miao, W., Wang, X., Kong, W., Xu, X., Wu, Y., Rui, Q., & La, H. (2018). Arabidopsis subtilase SASP is involved in the regulation of ABA signaling and drought tolerance by interacting with OPEN STOMATA 1. *Journal of Experimental Botany*, *69*(18), 4403–4417. https://doi.org/10.1093/jxb/ery205

Wang, Z., Ren, Z., Cheng, C., Wang, T., Ji, H., Zhao, Y., Deng, Z., Zhi, L., Lu, J., Wu, X., Xu, S., Cao, M., Zhao, H., Liu, L., Zhu, J., & Li, X. (2020). Counteraction of ABA-Mediated Inhibition of Seed Germination and Seedling Establishment by ABA Signaling Terminator in Arabidopsis. *Molecular Plant*, *13*(9), 1284–1297. https://doi.org/10.1016/j.molp.2020.06.011

Wu, Q., Zhang, X., Peirats-Llobet, M., Belda-Palazon, B., Wang, X., Cui, S., Yu, X., Rodriguez, P. L., & An, C. (2016). Ubiquitin ligases RGLG1 and RGLG5 regulate abscisic acid signaling by controlling the turnover of phosphatase PP2CA. *Plant Cell*, *28*(9), 2178–2196. https://doi.org/10.1105/tpc.16.00364

Xu, X., Wan, W., Jiang, G., Xi, Y., Huang, H., Cai, J., Chang, Y., Duan, C. G., Mangrauthia, S. K., Peng, X., Zhu, J. K., & Zhu, G. (2019). Nucleocytoplasmic Trafficking of the Arabidopsis WD40 Repeat Protein XIW1 Regulates ABI5 Stability and Abscisic Acid Responses. *Molecular Plant*, *12*(12), 1598–1611. https://doi.org/10.1016/j.molp.2019.07.001

Yang, J., He, H., He, Y., Zheng, Q., Li, Q., Feng, X., Wang, P., Qin, G., Gu, Y., Wu, P., Peng, C., Sun, S., Zhang, Y., Wen, M., Chen, R., Zhao, Y., & Xu, T. (2021). TMK1-based auxin signaling regulates abscisic acid responses via phosphorylating ABI1/2 in Arabidopsis. *Proceedings of the National Academy of Sciences*, *118*(24), e2102544118. https://doi.org/10.1073/pnas.2102544118

Yu, F., Lou, L., Tian, M., Li, Q., Ding, Y., Cao, X., Wu, Y., Belda-Palazon, B., Rodriguez, P. L., Yang, S., & Xie, Q. (2016). ESCRT-I Component VPS23A Affects ABA Signaling by Recognizing ABA Receptors for Endosomal Degradation. *Molecular Plant*, *9*(12), 1570–1582. https://doi.org/10.1016/j.molp.2016.11.002

Yu, F., Qian, L., Nibau, C., Duan, Q., Kita, D., Levasseur, K., Li, X., Lu, C., Li, H., Hou, C., Li, L., Buchanan, B. B., Chen, L., Cheung, A. Y., Li, D., & Luan, S. (2012). FERONIA receptor kinase pathway suppresses abscisic acid signaling in Arabidopsis by activating ABI2 phosphatase. *Proceedings of the National Academy of Sciences of the United States of America*, *109*(36), 14693–14698. https://doi.org/10.1073/pnas.1212547109

Yu, Z., Zhang, D., Xu, Y., Jin, S., Zhang, L., Zhang, S., Yang, G., Huang, J., Yan, K., Wu, C., Zheng, C., & Murphy, A. (2019). CEPR2 phosphorylates and accelerates the degradation of PYR/PYLs in Arabidopsis. *Journal of Experimental Botany*, *70*(19), 5457–5469. https://doi.org/10.1093/jxb/erz302

Zhang, L., Li, X., Li, D., Sun, Y., Li, Y., Luo, Q., Liu, Z., Wang, J., Li, X., Zhang, H., Lou, Z., & Yang, Y. (2018). CARK1 mediates ABA signaling by phosphorylation of ABA receptors. *Cell Discovery*, *4*(1), 1–10. https://doi.org/10.1038/s41421-018-0029-y

Zhao, J., Zhao, L., Zhang, M., Zafar, S. A., Fang, J., Li, M., Zhang, W., & Li, X. (2017). Arabidopsis E3 ubiquitin ligases PUB22 and PUB23 negatively regulate drought tolerance by targeting ABA receptor PYL9 for degradation. *International Journal of Molecular Sciences*, *18*(9), 8–10. https://doi.org/10.3390/ijms18091841

Zhou, X., Hao, H., Zhang, Y., Bai, Y., Zhu, W., Qin, Y., Yuan, F., Zhao, F., Wang, M., Hu, J., Xu, H., Guo, A., Zhao, H., Zhao, Y., Cao, C., Yang, Y., Schumaker, K. S., Guo, Y., & Xie, C. G. (2015). SOS2-LIKE PROTEIN KINASE5, an SNF1-RELATED PROTEIN KINASE3-Type Protein Kinase, Is Important for Abscisic Acid Responses in Arabidopsis through Phosphorylation of ABSCISIC ACID-INSENSITIVE5. *Plant Physiology*, *168*(2), 659 LP – 676. https://doi.org/10.1104/pp.114.255455

Zhu, S.-Y., Yu, X.-C., Wang, X.-J., Zhao, R., Li, Y., Fan, R.-C., Shang, Y., Du, S.-Y., Wang, X.-F., Wu, F.-Q., Xu, Y.-H., Zhang, X.-Y., & Zhang, D.-P. (2007). Two calcium-dependent protein kinases, CPK4 and CPK11, regulate abscisic acid signal transduction in Arabidopsis. *The Plant Cell*, *19*(10), 3019–3036. https://doi.org/10.1105/tpc.107.050666
